# Treatment with proteasome inhibitor bortezomib decreases organic anion transporting polypeptide (OATP) 1B3-mediated transport in a substrate-dependent manner

**DOI:** 10.1371/journal.pone.0186924

**Published:** 2017-11-06

**Authors:** Khondoker Alam, Taleah Farasyn, Alexandra Crowe, Kai Ding, Wei Yue

**Affiliations:** 1 Department of Pharmaceutical Sciences, College of Pharmacy, University of Oklahoma Health Sciences Center, Oklahoma City, OK, United States of America; 2 Department of Biostatistics and Epidemiology, College of Public Health, University of Oklahoma Health Sciences Center, Oklahoma City, OK, United States of America; University of Kentucky, UNITED STATES

## Abstract

OATP1B1 and OATP1B3 mediate hepatic uptake of many drugs (e.g., statins) and can mediate transporter-mediated drug-drug-interactions (DDIs). Bortezomib is the first-in-class proteasome inhibitor drug approved by the U. S. Food and Drug Administration for the treatment of multiple myeloma. The potential of bortezomib to cause OATP-mediated DDIs has not been assessed. The current study investigated the involvement of the ubiquitin-proteasome system (UPS) in OATP1B1 and OATP1B3 degradation and determined the effects of proteasome inhibitors on OATP1B1- and OATP1B3-mediated transport. Co-immunoprecipitation of FLAG-OATP1B1/1B3 and HA-ubiquitin was observed in human embryonic kidney (HEK) 293 cells co-transfected with FLAG-tagged OATP1B1/OATP1B3 and hemagglutinin (HA)-tagged ubiquitin, suggesting that OATP1B1 and OATP1B3 can be ubiquitin-modified. Although blocking proteasome activity by bortezomib treatment (50 nM, 7 h) increased the endogenous ubiquitin-conjugated FLAG-OATP1B1 and FLAG-OATP1B3 in HEK293-FLAG-OATP1B1 and–OATP1B3 cells, such treatment did not affect the total protein levels of OATP1B1 and OATP1B3, suggesting that the UPS plays a minor role in degradation of OATP1B1 and OATP1B3 under current constitutive conditions. Pretreatment with bortezomib (50–250 nM, 2–7 h) significantly decreased transport of [^3^H]CCK-8, a specific OATP1B3 substrate, in HEK293-OATP1B3 and human sandwich-cultured hepatocytes (SCH). However, bortezomib pretreatment had negligible effects on the transport of [^3^H]E_2_17βG and [^3^H]pitavastatin, dual substrates of OATP1B1 and OATP1B3, in HEK293-OATP1B1/1B3 cells and/or human SCH. Compared with vehicle control treatment, bortezomib pretreatment significantly decreased the maximal transport velocity (V_max_) of OATP1B3-mediated transport of CCK-8 (92.25 ± 14.2 vs. 133.95 ± 15.5 pmol/mg protein/min) without affecting the affinity constant (K_m_) values. Treatment with other proteasome inhibitors MG132, epoxomicin, and carfilzomib also significantly decreased OATP1B3-mediated [^3^H]CCK-8 transport. In summary, the current studies for the first time report ubiquitination of OATP1B1 and OATP1B3 and the apparent substrate-dependent inhibitory effect of bortezomib on OATP1B3-mediated transport. The data suggest that bortezomib has a low risk of causing OATP-mediated DDIs.

## Introduction

OATP1B1 and OATP1B3 are predominantly expressed on the basolateral membrane of hepatocytes under physiological conditions and mediate the hepatic uptake of many clinically important drugs, e.g., lipid-lowering statins, antibiotics, and anti-cancer drugs [1]. Drugs that are potent OATP inhibitors, e.g., cyclosporine [2], may cause clinically significant drug-drug interactions (DDIs) when prescribed with OATP substrates, such as statins. For example, increases in the systemic exposure of statins, statin-related myopathy, and even rhabdomyolysis have been observed when these drugs are co-administered with OATP inhibitors [3, 4]. OATP1B1 and OATP1B3 have overlapping substrates specificity; some substrates, such as statins [5, 6] and bromosulphophthalein [7], can be transported by both OATP1B1 and OATP1B3. However, some OATP1B3-specific substrates, such as the endogenous compound octapeptide cholecystokinin 8 (CCK-8), have been characterized [8].

The lysosome pathway and ubiquitin-proteasome system (UPS) are the major mechanisms through which proteins are degraded intracellularly [9]. We previously reported that the lysosome pathway is involved in OATP1B1 degradation, and that treatment with the lysosome inhibitor chloroquine decreases OATP1B1-mediated transport *in vitro* and is associated with increased statin-related myopathy in patients treated concurrently with chloroquine and metabolically stable statins [10]. In addition to being degraded through the lysosome [11], membrane proteins, including some membrane-transport proteins, can be degraded via the ubiquitin-proteasome system [12–14]. The degradation of a protein via the ubiquitin proteasome system involves the tagging of the protein by the attachment of multiple ubiquitin molecules to mediate its recognition and subsequent degradation through the proteasome [15]. Inhibition of proteasome activity by proteasome inhibitors has been associated with altered transport protein function [16, 17]. Currently, the ubiquitination of OATP1B1 and OATP1B3 and the effects of proteasome inhibition on their transport function have not been elucidated.

The proteasome inhibitor bortezomib is currently the front-line therapy for the treatment of newly diagnosed multiple myeloma patients and for patients with mantle cell lymphoma who have received at least one prior therapy [18]. The interaction of bortezomib with metabolizing enzymes has been characterized in the past decade. Bortezomib is a substrate of several cytochrome P450 isoenzymes [19], but only mildly inhibits CYP2C19 and CYP2C9; therefore, no major metabolism-mediated DDIs are anticipated [20]. Statins are widely prescribed for the treatment of hypercholesterolemia and hypertension, and are also reported to have beneficial effects in cancers, including multiple myeloma [21–23]. Since elevated systemic exposure to statins due to DDIs may cause severe side effects, determining the DDI potential and myopathy risk associated with bortezomib upon concurrent administration with OATP substrate statins has significant clinical relevance. To date, the potential of bortezomib to cause OATP-mediated DDIs has not been assessed.

The current studies were designed to determine the ubiquitination of OATP1B1 and OATP1B3 and to investigate the effects of proteasome inhibitors on OATP1B1- and OATP1B3-mediated transport. OATP1B1- and OATP1B3-expressing human embryonic kidney (HEK) 293 stable cell lines and the physiologically relevant human sandwich-cultured hepatocytes (SCH) model were used in our studies.

## Materials and methods

### Materials

Unlabeled cholecystokinin-8 (CCK-8), estradiol 17β-D-glucuronide (E_2_17βG), IGEPAL (NP-40), Hanks' Balanced Salt Solution (HBSS), dexamethasone, dimethyl sulfoxide (DMSO), Triton X-100, Dulbecco's Modified Eagle Medium (DMEM), fetal bovine serum (FBS), N-ethylmaleimide, trypsin-EDTA solution, antibiotic antimycotic solution, Dulbecco's Phosphate-Buffered Saline (DPBS), bromosulfophthalein (BSP), bovine serum albumin (BSA), and epoxomicin were purchased from Sigma-Aldrich (St. Louis, MO, USA). Carfilzomib was obtained from LC laboratories (Woburn, MA, USA). Insulin, minimum essential medium (MEM), nonessential amino acids (NEAA), L-glutamine, Geneticin, and penicillin-streptomycin were procured from Life Technologies (Grand Island, NY, USA). Matrigel and insulin/transferrin/selenium (ITS^+^ premix) were purchased from BD Biosciences (Bedford, MA). Cultrex^®^ poly-L-lysine was obtained from Trevigen, Inc. (Gaithersburg, MD, USA). Complete protease inhibitor cocktail tablets were purchased from Roche Diagnostics (Indianapolis, IN, USA). Bortezomib was procured from Toronto Research Chemicals (Toronto, Ontario, Canada). MG132 was purchased from EMD Millipore (Billerica, MA, USA). [^3^H]CCK-8 (specific activity 96 Ci/mmol) and [^3^H]E_2_17βG (specific activity 41.4 Ci/mmol) were obtained from Perkin Elmer Life Science (Waltham, MA, USA). [^3^H]pitavastatin (specific activity 5 Ci/mmol) and unlabeled pitavastatin were purchased from American Radiolabeled Chemicals (St. Louis, MO, USA). Bio-Safe II liquid scintillation mixture was obtained from Research Products International (Mt. Prospect, IL). All other reagents were procured from VWR International (Radnor, PA, USA).

### Constructs and transfection

The pCMV6 plasmid vectors expressing FLAG-tagged OATP1B1 (pCMV6-FLAG-OATP1B1) and 1B3 (pCMV6-FLAG-Myc-OATP1B3) were described previously [10, 24]. The pcDNA3-3XHA-Ub plasmid (HA-Ub) was obtained from Dr. Shunbing Ning [25]. All plasmids were confirmed by sequencing. HEK293 cells were seeded at a density of 2.5 x 10^5^ cells per well in 24-well culture plates. Twenty-four hours after seeding, the cells were transfected with pCMV6-FLAG-Myc-OATP1B3, pCMV6-FLAG-OATP1B1, HA-Ub, or empty vector control (pCMV6), either alone or in combination, using Lipofectamine 2000 (Invitrogen by Life Technologies) per the manufacturer’s instructions. Forty-eight hours post-transfection, cells were washed once with PBS buffer and were subjected to subsequent immunoprecipitation and immunoblot.

### Cell culture

The human embryonic kidney (HEK) 293 stable cell lines overexpressing OATP1B1 (HEK293-OATP1B1) and OATP1B3 (HEK293-OATP1B3) were provided by Dr. Dietrich Keppler [26, 27]. The HEK293 stable cell line overexpressing FLAG-tagged OATP1B1 (HEK293-FLAG-OATP1B1) was described previously [10]. The HEK293 stable cell line overexpressing FLAG-Myc-tagged OATP1B3 (HEK293-FLAG-OATP1B3) was established by transfection of pCMV6-FLAG-Myc-OATP1B3 plasmid vector, which was described previously, in HEK293 cells, followed by selection with Geneticin (600 μg/ml). All HEK293 stable cell lines were maintained in DMEM medium containing 10% FBS, 1% antibiotic and antimycotic solution, and 600 μg/ml Geneticin, and were cultured in a humidified atmosphere (95% O_2_, 5% CO_2_) at 37°C.

### Human sandwich-cultured hepatocytes (SCH)

Human hepatocytes were purchased from Triangle Research Labs, LLC (Research Triangle Park, NC, USA) and Life Technology (Carlsbad, CA, USA). The demographics of the human hepatocyte donors are listed in Table 1. Human SCH were cultured as described previously [28, 29]. In brief, cells were seeded in 24-well Biocoat™ culture plates at a density of 3.5 X10^5^ cells per well on day zero in phenol red-free DMEM containing 5% (v/v) FBS, 1% (v/v) MEM NEAA, 2 mM L-glutamine, penicillin-streptomycin (10 U/ml), 1 μM dexamethasone, and 4 μg/ml insulin. Six hours after seeding, when the cells were attached to the plate, cells were overlaid with Matrigel™ at a final concentration of 0.25 mg/ml in phenol red-free DMEM supplemented with 2 mM L-glutamine, 1% (v/v) MEM NEAA, 100 units/ml of penicillin-streptomycin solution, 0.1 μM dexamethasone, and 1% (v/v) ITS^+^ premix. Experiments were conducted on day one after 24 h of culture.

**Table 1:** Demographics of human hepatocyte donors.

Donors	Age	Gender	Race	BMI	Smoking	Alcohol use	Experiments
Hu1707	73	F	U	27.4	No	No	Fig 5C
GC4008	69	M	C	24.7	No	No	Fig 3A, S3A Fig
Hu1709	59	F	C	29.5	Yes	No	Figs 3A and 5C
HUM 4089	36	M	C	30	No	No	Figs 3A and 5C
HUM4133	45	F	C	20.7	U	Rare	Figs 5C and 7E
HUM4130	64	M	AA	23.6	Quit	No	Fig 3A and 3C, S3B Fig
HUM4140	21	M	C	32	Yes	Rare	Figs 3A, 3C and 7E
Hu1795	59	M	C	28.1	No	Rare	Fig 5C
HUM4161	42	M	C	25	No	Social	Figs 3A, 3C, 4B, 5C and 7E
HUM4190	26	M	C	22.4	No	No	Fig 4B
HUM4194	42	M	A	24	No	No	Fig 4B
HUM4070	22	F	C	23.3	No	No	Fig 5C
Hu1682	53	M	C	27.6	No	Yes	Fig 5C

BMI: body mass index

U, Unknown; C, Caucasian; AA, Africa American; A, Asian

### Immunoprecipitation and immunoblot

Immunoprecipitation and immunoblot assays were conducted similar to those published previously [10, 24]. In brief, whole cell lysates (WCL) were prepared by lysing the cells with lysis buffer containing 50 mM Tris/HCl (pH 7.4), 150 mM NaCl, 1 mM EDTA, 1% NP-40, 0.5% Na-deoxycholate, and Complete^TM^ protease inhibitor cocktail (Roche Diagnostics USA, Indianapolis, IN, USA). Protein concentrations of lysates were determined using BCA assay (Pierce Chemical, Rockford, IL, USA). Whole cell lysates (0.5 mg) in 500 μl of lysis buffer were first precleared by incubation with normal mouse IgG (Santa Cruz, Dallas, TX, USA) and 30 μL of protein A/G PLUS-Agarose (Santa Cruz, Dallas, TX, USA) at 4°C for 30 min, with gentle rotation. After centrifugation, the supernatant was incubated with 5 μg of mouse monoclonal anti-FLAG M2 (F1804, Sigma, St. Louis, MO, USA) or 1 μg mouse monoclonal anti-HA (Santa Cruz, SC 7392) antibody at 4°C for 1 h. The immunocomplexes were then adsorbed by protein A/G-agarose beads at 4°C overnight. After washing four times with lysis buffer, the immunocomplexes were eluted from the protein A/G-agarose beads by boiling with 2X Laemmli buffer for 5 min at 100°C. In order to avoid protein aggregation, whole cell lysates for immunoblot were not boiled, unless stated otherwise. Whole cell lysates and eluted immunocomplexes were resolved through sodium dodecylsulfate–10% polyacrylamide gel electrophoresis (SDS-PAGE). After transferring the protein to the membrane, the blot was blocked with 5% milk in Tris-buffered saline with 0.05% Tween (TBST) and was incubated at room temperature (RT) for 2 h or overnight at 4°C with the following antibodies: FLAG monoclonal antibody (M2; 1:4000; Sigma, St. Louis, MO, USA), HA (1:1000 dilution; Santa Cruz, Dallas, TX, USA), USA, β-actin monoclonal antibody (1:5000 dilution; Sigma, St. Louis, MO, USA), custom-generated rabbit polyclonal antibodies for OATP1B1 (1:2000) and OATP1B3 (1:1000) [10], and mouse monoclonal ubiquitin antibody (clone P4D1,1:1000, Santa Cruz, Dallas, TX, USA). After rinsing with TBST, the blots were incubated with the appropriate horseradish peroxidase-conjugated secondary antibodies (1:5000; Santa Cruz, Dallas, Texas) for 1 h at room temperature. After incubation with the SuperSignal West Dura chemiluminescent substrate (Pierce, Rockford, IL), images were captured using a Bio-Rad ChemiDoc XRS imaging system (Bio-Rad Laboratories, Hercules, CA). All immunoblot images shown in the current manuscript were captured before saturation and adjusted for contrast and brightness at linear range for optimal visualization. Densitometry analyses were performed using Image Lab v4.1 software (Bio-Rad Laboratories, Hercules, CA).

### Transport studies

HEK293-OATP1B1 and -OATP1B3 cells were seeded at a density of 1–1.2 x 10^5^ cells/well in 24-well culture plates and were cultured to confluence. HEK293 stable cell lines or human SCH were either pre-incubated with culture medium containing testing compounds or vehicle control (pre-incubation) before beginning the transport studies, or remained un-treated (co-incubation). Substrate concentrations used in the current studies were all below the reported K_m_ values for respective transporters of OATP1B1 and OATP1B3 [30]. Accumulation times of OATP1B1-mediated transport of [^3^H]E17βG (1 μM, 2 min) and [^3^H]pitavastatin (1 μM, 0.5 min) and OATP1B3-mediated transport of [^3^H]CCK-8 (1 μM, 3 min) and [^3^H]E17βG (1 μM, 2 min) were at the linear uptake range published previously [10, 29–31]. The linear uptake range of OATP1B3-mediated [3H]pitavastatin (1 μM) was determined in the current studies. To perform the uptake assay, cells were rinsed with pre-warmed (37°C) HBSS buffer (pH 7.4) three times, and then incubated with HBSS buffer containing [^3^H]CCK-8 (1 μM, 3 min), [^3^H]E_2_17βG (1 μM, 2 min) or [^3^H]pitavastatin (1 μM, 0.5 min) in the absence or presence of bortezomib, rifampicin or bromosulfophthalein (BSP), as indicated in the figure legends. Rifampicin and bromosulfophthalein are potent OATP inhibitors [32, 33] and served as the positive controls [29, 34]. At the end of the co-incubation, the buffer was aspirated rapidly and the cells were rinsed with ice-cold HBSS three times before lysing with Triton-X 100 (0.5% v/v) in DPBS. An aliquot of the lysate was subjected to liquid scintillation counting (LS6500 scintillation counter, Beckman Coulter, Brea, CA). Substrate accumulation was normalized to the protein concentration determined by BCA assay (Pierce Chemical, Rockford, IL) and corrected for nonspecific binding of the substrate by subtracting the accumulation values observed after performing the same assay in a non-overlaid poly-L-lysine coated blank plate for the uptake studies in stable cell lines and a Matrigel™ overlaid blank plate for the human SCH studies. To determine the long-lasting inhibitory effect of bortezomib on OATP1B3-mediated [^3^H]CCK-8 transport, HEK293-OATP1B3 cells were pre-incubated with vehicle control or bortezomib (50 nM, 2 h). At the end of pre-incubation, the culture medium was aspirated. Cells were washed three times with fresh culture media, and then incubated with fresh culture media without bortezomib for the indicated time up to 24 h. To determine the effect of bortezomib on [^3^H]CCK-8 transport kinetic parameters, the maximal transport velocity (V_max_) and the affinity constant (K_m_), [^3^H]CCK-8 accumulation (0.1–40 μM, 3 min) were determined in HEK293-OATP1B3 cells after pretreatment with bortezomib (50 nM, 7 h) or vehicle control. The V_max_ and K_m_ values of CCK-8 transport were estimated by fitting the Michaelis–Menten equation below to the data using GraphPad Prism v.7.0 (GraphPad Software, La Jolla, CA, USA), where v is the CCK-8 transport velocity and S is the CCK-8 concentration.

$$v=\frac{V_{max}*S}{K_{m}+S}$$

### Surface biotinylation assay

Experiments were conducted similarly to those published previously, with slight modifications [35]. All steps were conducted at 4°C or on ice, and with ice-cold buffers. HEK293-OATP1B3 cells were seeded on 24-well plates at a density of 1.2 x 10^5^ cells per well, cultured for 48 h, and then treated with bortezomib (50 nM) or vehicle control in culture medium for 7 h at 37°C. At the end of treatment, the cells were incubated in DPBS (with calcium and magnesium) containing 1 mg/ml of sulfo-NHS-SS-biotin (Pierce Chemical, Rockford, IL) for 1 h at 4°C. Subsequently, the cells were washed three times with ice-cold DPBS (with calcium and magnesium) containing 100 mM glycine. The cells were then lysed with lysis buffer containing 10 mM Tris (pH 7.5), 150 mM NaCl_2_, 1 mM EDTA, 0.1% SDS, 1% TritonX-100, and Complete™ protease inhibitor cocktail, sonicated, and the supernatant proteins (200 μg) were incubated with NeutrAvidin beads (Pierce, Rockford, IL) overnight with gentle rocking. After washing five times with ice-cold lysis buffer, the beads were incubated with 2X Laemmli buffer for 1 h at room temperature to elute the protein. The supernatant was then resolved on 10% SDS-PAGE (Bio-Rad Laboratories, Inc., Hercules, CA, USA) and was subsequently immunoblotted with OATP1B3 (1:500 dilution) and Na-K-ATPase (1:8000 dilution; Abcam, Cambridge, MA, USA) antibodies. Na-K-ATPase was used as a loading control for surface protein levels of OATP1B3 [36, 37]. The blots were also probed with a GAPDH antibody (1:1000 dilution, Santa Cruz, Dallas, TX, USA) to determine whether the surface fraction had intracellular protein contamination, similar to previously published. After incubation with the respective secondary antibodies, IRDye 800CW goat anti-rabbit (1:20000), or IRDye 680RD goat anti-mouse (1:20000) antibodies, immunoblot images were captured before saturation using an Odyssey CLx Imaging System (LI-COR Biotechnology, Lincoln, NE) according to the manufacturer’s instructions. Densitometry was conducted using the Image Studio software (LI-COR Biotechnology, Lincoln, NE, USA).

### Lactate dehydrogenase (LDH) cytotoxicity assay

Experiments were conducted similar to those published previously [38]. After treatment with bortezomib or vehicle control, the cell culture media was assayed for lactate dehydrogenase (LDH) activity with a cytotoxicity detection kit (Roche Diagnostics GmbH, Mannheim, Germany), according to the manufacturer's instructions. Triton-X (2%)-treated and non-treated cells served as the 100% cytotoxicity positive control and negative control, respectively.

### Data analysis

As indicated in the figure legends, fold changes and associated standard errors (SEs) were estimated by linear mixed effects models with a fixed effect (treatment time or group) and a random effect (experiment date or hepatocyte donor), allowing for treatment time/group-specific variation, similar to what has been published previously [10, 29]. In the case of multiple comparisons, p-values were adjusted based on the Bonferroni’s method within the linear mixed effects models. A two-sided p-value of <0.05 defines statistical significance. SAS software (version 9.3, Cary, NC) was used for statistical analyses.

## Results

### Ubiquitination of OATP1B1 and OATP1B3

**Fig 1:**
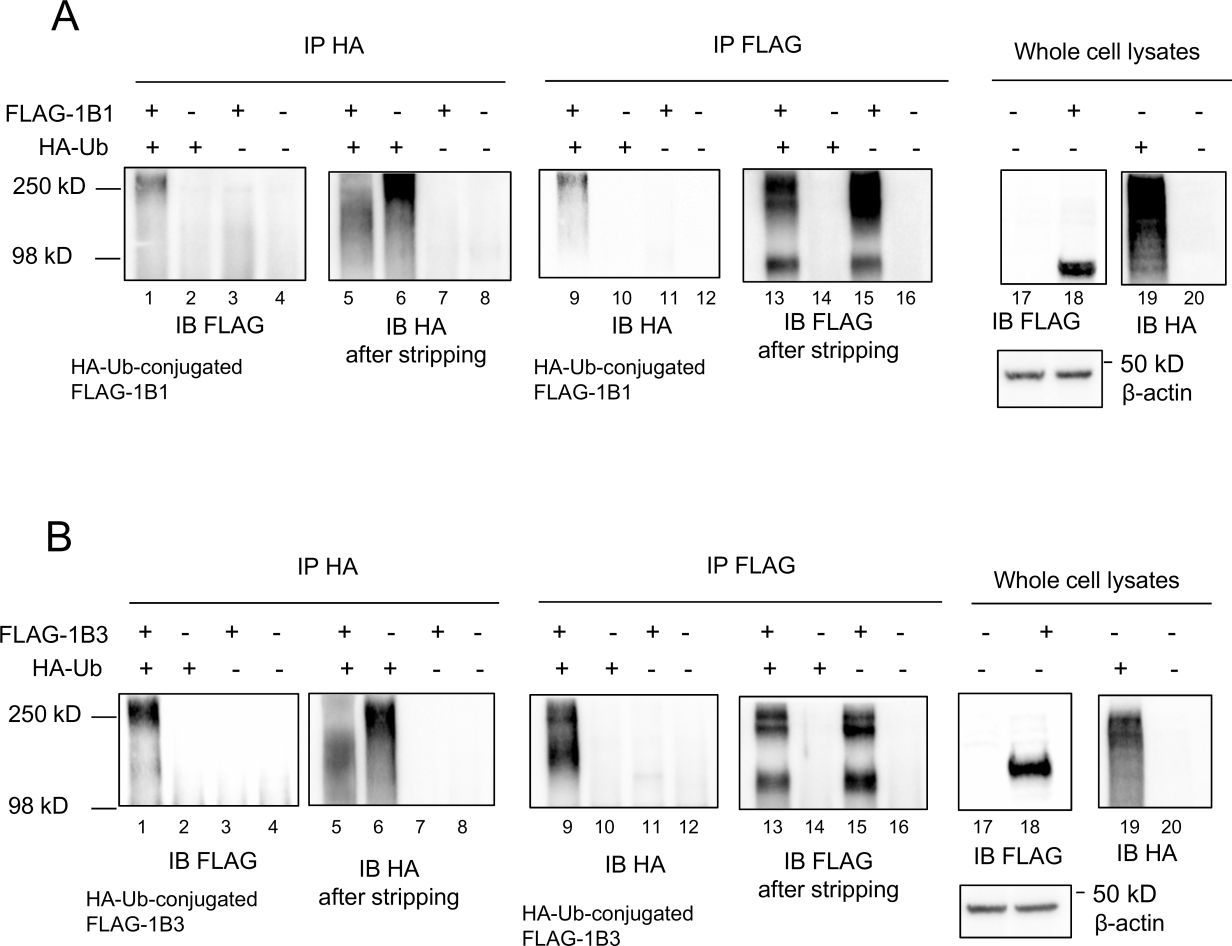
Co-immunoprecipitation of FLAG-OATP1B1/OATP1B3 with HA-Ub. pCMV6-FLAG-OATP1B1 (A) or pCMV6-FLAG-OATP1B3 (B) vectors were transfected into HEK293 cells, either alone or in combination with HA-Ub expression vector. Transfection with empty vector pCMV6 served as the vehicle control in all experiments. Whole cell lysates (WCL) were subjected to immunoprecipitation (IP) with HA or FLAG antibody, followed by immunoblotting (IB) with FLAG or HA antibody, respectively. After stripping, the same blot was re-probed with HA or FLAG antibody, as indicated in the figure. Immunoblots of HA and FLAG were also conducted using WCL with β-actin serving as the loading control. Representative immunoblot images from n = 3 independent experiments are shown.

To determine whether OATP1B1 and OATP1B3 could be modified by ubiquitination, co-immunoprecipitation (IP) of FLAG-tagged OATP1B1/OATP1B3 with HA-tagged ubiquitin (HA-Ub) was performed in HEK293 cells that were transiently transfected with vectors expressing FLAG-OATP1B1 or FLAG-OATP1B3, either alone or in combination with HA-ubiquitin, similar to what has been published previously [39]. After IP with HA antibody and immunoblotting (IB) with FLAG antibody, the HA-ubiquitin-conjugated FLAG-OATP1B1 (Fig 1A, lane 1) and FLAG-OATP1B3 (Fig 1B, lane 1) were readily detected when HA-Ub was co-expressed with FLAG-OATP1B1 or FLAG-OATP1B3, respectively. Similarly, after IP with FLAG antibody and immunoblotting with HA antibody, the HA-Ub that was conjugated to FLAG-OATP1B1 (Fig 1A, lane 9) and FLAG-OATP1B3 (Fig 1B, lane 9) was readily detected only when HA-Ub was co-expressed with FLAG-OATP1B1 or FLAG-OATP1B3, respectively.

To confirm the successful immunoprecipitation of HA and FLAG, the same membrane was stripped and re-probed with FLAG or HA antibody, respectively (Fig 1A and 1B, after stripping). The HA and FLAG signals were specifically detected in HA-Ub- and FLAG-OATP1B1/1B3-transfected cells after IP with HA and FLAG, respectively, but not in the vector control-transfected cells. The expression of HA-Ub, FLAG-OATP1B1, and FLAG-OATP1B3 were also confirmed in whole cell lysates by immunoblotting with HA and FLAG antibodies, with β-actin as the loading control (Fig 1A and 1B, whole cell lysates).

### Effects of bortezomib treatment on ubiquitination and protein levels of OATP1B1 and OATP1B3

We determined the effects of bortezomib treatment on the levels of ubiquitin-conjugated OATP1B1 and OATP1B3. Since our custom-generated OATP1B1 and OATP1B3 antibodies [10, 24] have not been validated for immunoprecipitation, the HEK293-FLAG-OATP1B1 [10] and the newly established HEK293–FLAG-OATP1B3 stable cell lines were used for this purpose, as FLAG-tagged OATP1B1 and -1B3 proteins can be readily immunoprecipitated using a FLAG antibody. As shown in S1 Fig, immunoblotting of OATP1B3 (S1A Fig) and FLAG antibodies (S1B Fig) specifically detected FLAG-OATP1B3 expression in the HEK293-FLAG-OATP1B3 stable cell line, but not in the negative control HEK293-Mock cell line. Accumulation of [^3^H]CCK-8 (1 μM, 3 min) in HEK293-FLAG-OATP1B3 cells was ~100 fold higher than in the negative control HEK293-Mock cells (S1C Fig).

**Fig 2:**
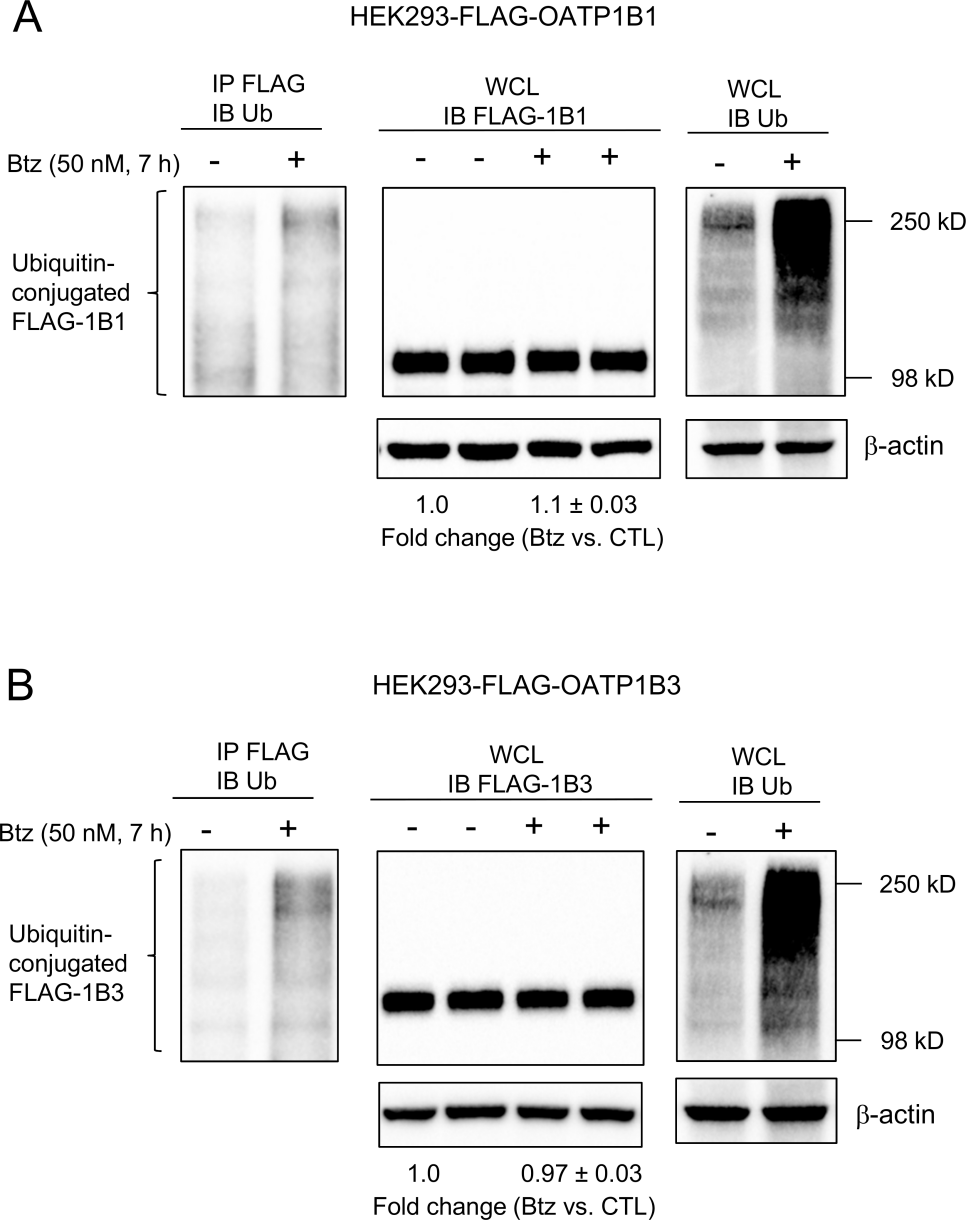
Effects of bortezomib on the ubiquitination and total protein levels of FLAG-OATP1B1 and FLAG-OATP1B3 in HEK293-FLAG-OATP1B1 and HEK293-FLAG-OATP1B3 cell lines. HEK293-FLAG-OATP1B1 and–FLAG-OATP1B3 stable cell lines were seeded at a density of 3x10^6^ cells per 100-mm^2^ dish. After culturing for 48 h, cells were treated with bortezomib (Btz) (50 nM, 7 h) or vehicle control (CTL). Whole cell lysates (WCL) (500 μg) from HEK293-FLAG-OATP1B1 (A, left panel) and HEK293-FLAG-OATP1B3 (B, left panel) were subjected to immunoprecipitation (IP) with FLAG antibody, followed by immunoblotting with ubiquitin antibody. Immunoblots of FLAG (A and B, middle panels) and ubiquitin (right panels of A and B) were conducted using whole cell lysates (50 μg) of HEK293-FLAG-OATP1B1 (A) and HEK293-FLAG-OATP1B3 (B) cells treated with bortezomib (50 nM, 7 h) or vehicle control. β-actin served as the loading control. FLAG-OATP1B1 and FLAG-OATP1B3 protein levels determined by densitometry were normalized to levels of β-actin. Fold changes of total protein levels in bortezomib-treated cells vs. CTL were expressed as mean ± SD. Representative images from n = 3 independent experiments are shown.

The FLAG-OATP1B1 and FLAG-OATP1B3 proteins that were ubiquitin-conjugated were determined via immunoprecipitation with FLAG antibody, followed by immunoblot with ubiquitin antibody. After bortezomib treatment (50 nM, 7 h), the total ubiquitin-conjugated proteins were markedly increased in HEK293-FLAG-OATP1B1 (Fig 2A, right panel) and -OATP1B3 cells (Fig 2B, right panel), respectively, compared with vehicle control treatment, suggesting efficient proteasome inhibition by bortezomib treatment. Compared with vehicle control treatment, more ubiquitin-conjugated FLAG-OATP1B1 and FLAG-OATP1B3 proteins were detected in bortezomib-treated HEK293-FLAG-OATP1B1 (Fig 2A, left panel) and HEK293-FLAG-OATP1B3 cells (Fig 2B, left panel), respectively. The total protein levels of FLAG-OATP1B1 (Fig 2A, middle panel) and FLAG-OATP1B3 (Fig 2B, middle panel) in bortezomib-treated cells were 1.1 ± 0.03 and 0.97 ± 0.03 fold of vehicle control treatment. Similarly, in HEK293-OATP1B1 and HEK293-OATP1B3 stable cell lines, bortezomib treatment (50 and 250 nM, 2 and 7 h) did not affect protein levels of OATP1B1 and OATP1B3, respectively (S2A–S2D Fig). Treatment with bortezomib (50 and 250 nM, 2 and 7 h) also efficiently inhibited the proteasome activity in HEK293-OATP1B1 and HEK293-OATP1B3 cells, as indicated by markedly increased ubiquitin-conjugated proteins (S2E and S2F Fig).

### Effects of bortezomib on endogenous OATP1B1 and OATP1B3 protein levels and on total ubiquitin-conjugated proteins in human SCH

**Fig 3:**
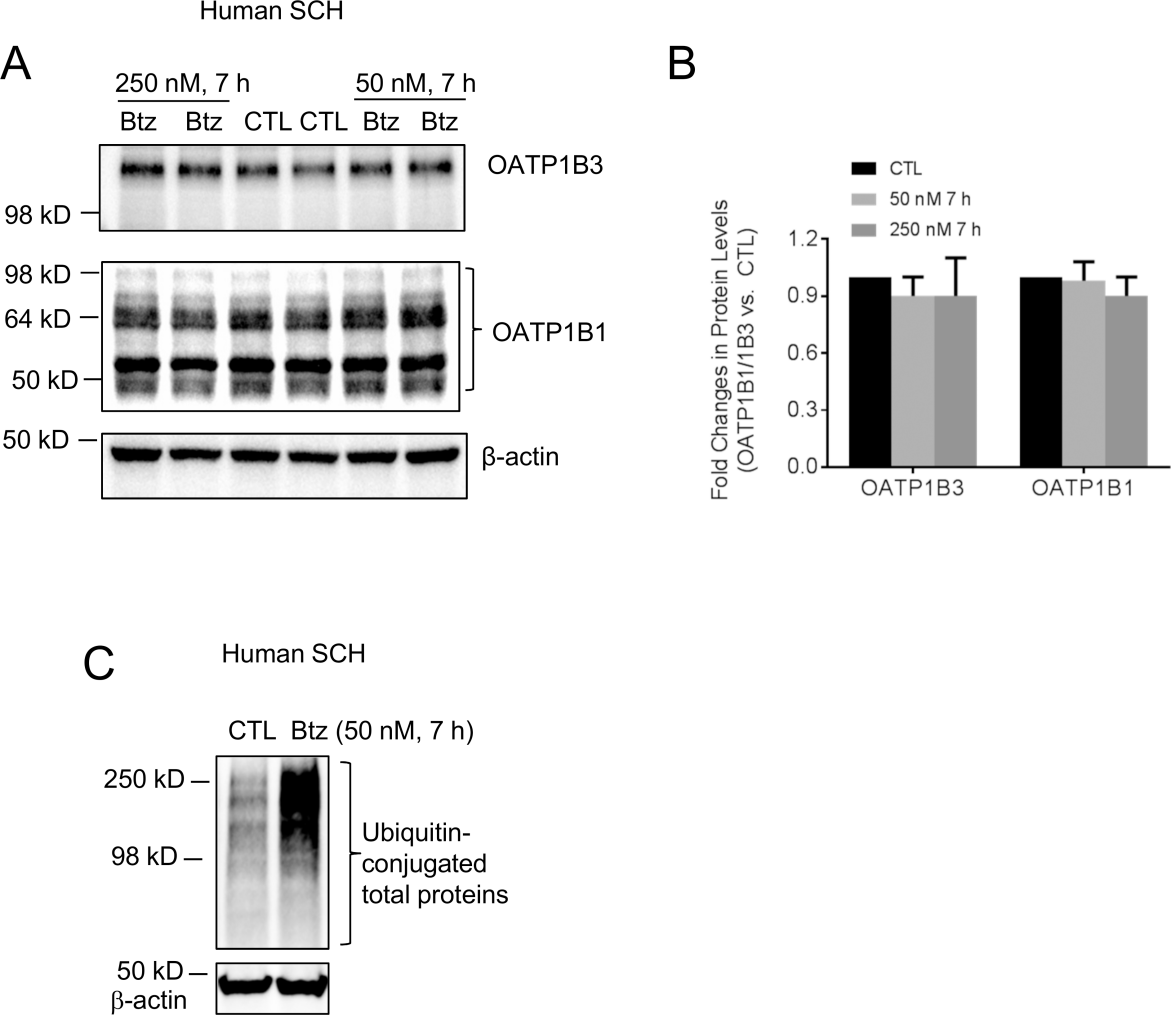
Effects of bortezomib on total OATP1B1 and OATP1 B3 protein levels and total ubiquitin-conjugated proteins in human SCH. Human SCH were cultured as described in the Materials and Methods section. (A) Immunoblot of OATP1B3 and OATP1B1 in whole cell lysates of human SCH that were treated with bortezomib (Btz) (50 and 250 nM) or vehicle control (CTL). β-actin served as the loading control. Representative images are shown from n = 3 and 4 donors for 50 and 250 nM treatment, respectively. (B) Fold changes of OATP1B1 and OATP1B3 protein levels. Densitometry of OATP1B1 and OATP1B3 protein levels was normalized to that of β-actin. Fold changes of total protein levels of OATP1B1 and OATP1B3 in bortezomib-treated cells vs. CTL were expressed as mean ± SD n = 3 and 4 donors for 50 and 250 nM treatment, respectively. (C) Immunoblot of ubiquitin in whole cell lysates of human SCH treated with bortezomib (Btz) (50 nM, 7 h) or vehicle control. β-actin served as the loading control. Representative images are shown from n = 3 donors.

Effects of bortezomib treatment on total protein levels of endogenous OATP1B1 and OATP1B3 were determined in human SCH. After pretreatment with bortezomib for 7 h at 50 and 250 nM, protein levels of OATP1B3 were 0.9 ± 0.1 and 0.9 ± 0.2 folds of vehicle control treatment, while protein levels of OATP1B1 were 0.98 ± 0.1 and 0.90 ± 0.1 folds of vehicle control treatment (Fig 3A and 3B). The total ubiquitin-conjugated proteins were markedly increased after bortezomib treatment (50 nM, 7 h) in human SCH (Fig 3C), confirming efficient inhibition of proteasome activity by bortezomib treatment. Representative images of OATP1B1 and OATP1B3 immunoblots overlaid with molecular weight markers are shown in the supplementary results (S3 Fig). Similar to what was observed from whole cell lysates of transporter-expressing stable cell lines (Fig 2 middle panels), following bortezomib treatment, no higher molecular weight bands of OAPT1B1 or OATP1B3 appeared in human SCH whole cell lysates (S3 Fig). The molecular weight of OATP1B3 observed in human SCH (Fig 3A and S3 Fig) is similar to that described previously in human hepatocytes and human liver tissues [40], and is also similar to OATP1B3-expressing HEK293 stable cell lines (Fig 2B middle panel and S3A Fig, right panel). The molecular weights of OATP1B1 in human SCH are at approximately 98 kD, 64 kD and 50 kD. These molecular weights of OATP1B1 were described previously in primary human hepatocytes [10, 40, 41], human basolateral membrane [27] and human liver tissues [40]. The ~98 kD molecular weight band of OATP1B1 in human SCH (Fig 3A and S3 Fig) is at a similar size as in the transporter-expressing stable cell line (Fig 2A middle panel).

### Effects of lysosome inhibitor chloroquine on total protein levels of OATP1B3

**Fig 4:**
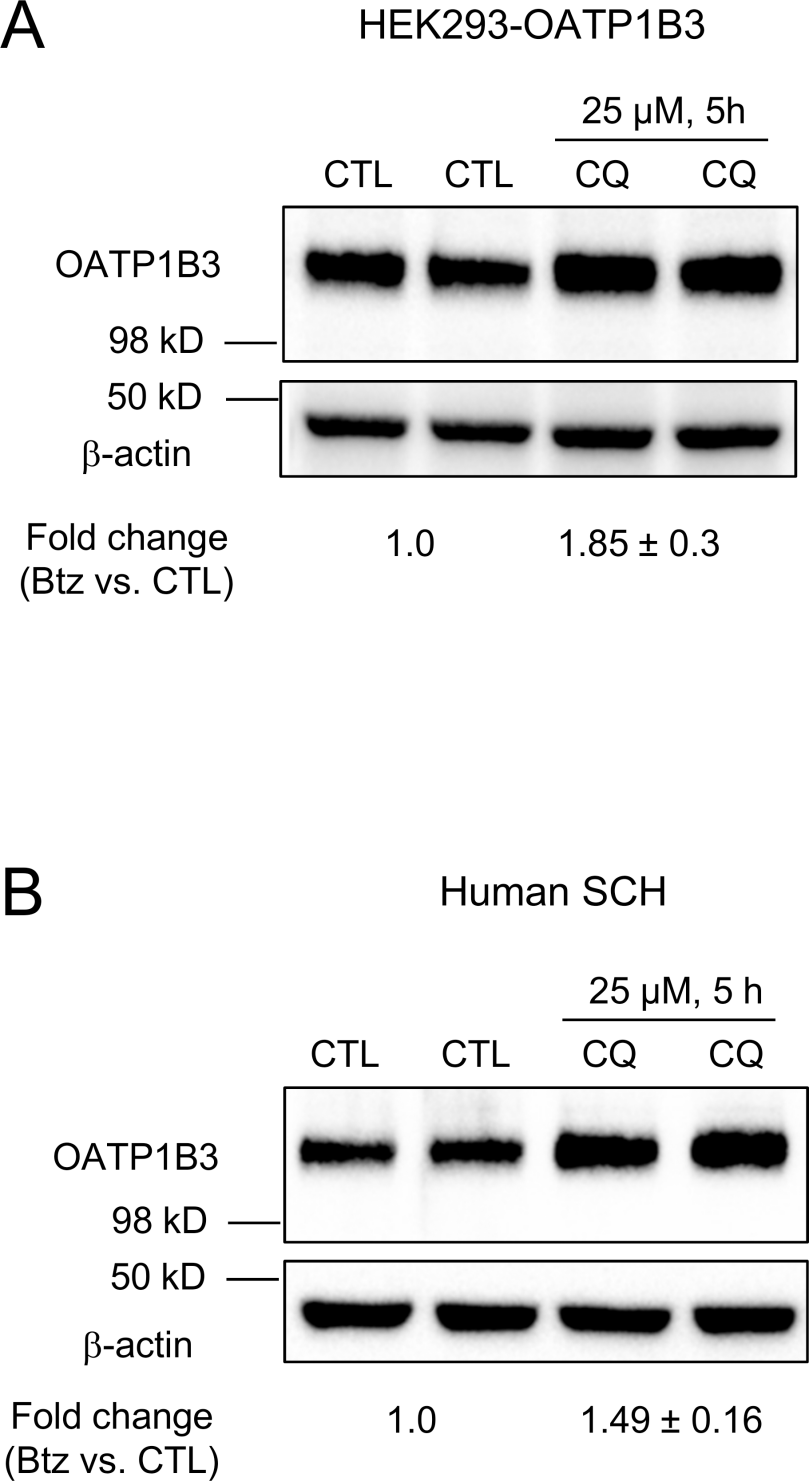
Effects of lysosome inhibitor chloroquine on total protein levels of OATP1B3 in HEK293-OATP1B3 cells and human SCH. HEK293-OATP1B3 cells were seeded in 24-well plates at a density of 1.2 x 10^5^ cells/well and were cultured for 48 h prior to treatment. Human SCH were cultured as described in the Materials and Methods section. Cells were pretreated with chloroquine (CQ) (25 μM, 5 h) or vehicle control (CTL) prior to immunoblotting for OATP1B3 in HEK293-OATP1B3 cells (A) and in day 1 human SCH (B). β-actin served as the loading control in both A and B. OATP1B3 protein levels determined by densitometry were normalized to levels of β-actin. Fold changes of total protein levels of OATP1B3 in chloroquine (CQ) -treated cells vs. vehicle control treatment (CTL) were expressed as mean ± SD (n = 3).

We previously reported that total protein levels of OATP1B1 were increased in a HEK293-OATP1B1 stable cell line and in human SCH after treatment with lysosome inhibitor chloroquine [10]. In the current studies, the effects of chloroquine treatment on total protein levels of OATP1B3 were determined in the HEK293-OATP1B3 stable cell line and in human SCH. Treatment with chloroquine (25 μM, 5 h) increased OATP1B3 protein levels to 1.85 ± 0.3 (mean ± SD, n = 3; Fig 4A) and 1.49 ± 0.16 (mean ± SD, n = 3 donors; Fig 4B) fold of vehicle control in HEK293-OATP1B3 cells and human SCH, respectively.

### Effects of bortezomib on OATP1B3-mediated transport in HEK293-OATP1B3 cells and in human SCH

**Fig 5:**
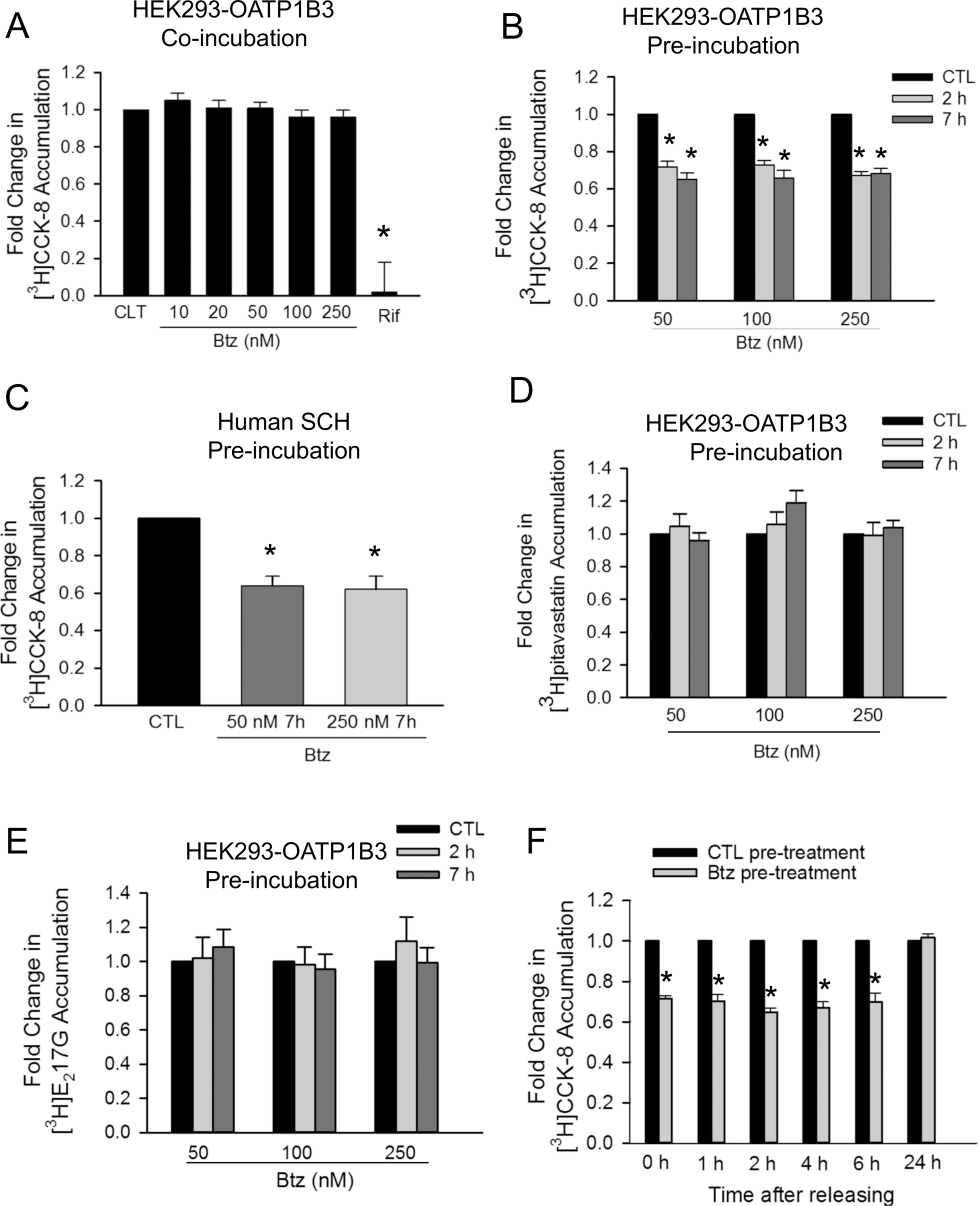
Effects of bortezomib on OATP1B3-mediated transport. (A) Model-estimated fold change and associated SE of [^3^H]CCK-8 accumulation (1 μM, 3 min) in the presence of 10–250 nM bortezomib (Btz) or 25 μM rifampicin (Rif) vs. control (CTL) in HEK293-OATP1B3 cells without any pre-incubation (Co-incubation). (B) Model-estimated fold change and associated SE of [^3^H]CCK-8 accumulation (1 μM, 3 min) in HEK293-OATP1B3 cells pretreated with bortezomib (Btz) vs. vehicle CTL at each indicated pretreatment concentration and time (Pre-incubation). After pretreatment, cells were washed three times with the HBSS buffer, and the [^3^H]CCK-8 accumulation was determined in the absence of bortezomib. (C) Model-estimated fold change and associated SE of [^3^H]CCK-8 accumulation (1 μM, 3 min) in human SCH pretreated with bortezomib (Btz) (50 and 250 nM, 7 h) vs. vehicle CTL. After pretreatment, cells were washed three times with the HBSS buffer, and the [^3^H]CCK-8 accumulation was determined in the absence of bortezomib. Model-estimated fold change and associated SE of OATP1B3-mediated [^3^H]pitavastatin (1 μM, 1 min) (D) and [^3^H]E_2_17βG accumulation (1 μM, 2 min) (E) in bortezomib pretreatment vs. vehicle CTL at each indicated pretreatment concentration and time (Pre-incubation). In D and E, HEK293-OATP1B3 and HEK293-Mock cells were pretreated with vehicle control (CTL) or bortezomib at the indicated concentrations and time. After washing with the HBSS buffer, OATP1B3-mediated [^3^H]pitavastatin (D) and [^3^H]E_2_17βG accumulation (E) was determined by subtracting the values determined in the HEK293-Mock cells from those in HEK293-OATP1B3 cells. (F) Model-estimated fold change and associated SE in [^3^H]CCK-8 accumulation (1 μM, 3 min) vs. CTL. Cells were pre-incubated with bortezomib-free (CTL) or 50 nM bortezomib-containing media for 2 h. At the end of pre-incubation, the culture medium was removed. After washing, CTL- and bortezomib-pretreated cells were cultured in bortezomib-free medium for the indicated time duration. [^3^H]CCK-8 (1 μM, 3 min) accumulation was determined at the indicated time points after washing three times (*n* = 3 in triplicate). A generalized linear mixed model was fit to the data in A-F as described in the “Materials and Methods” (n = 3 for A, D-F; n = 6 for B; n = 5 for C; all experiments were performed in triplicate). To account for multiple comparisons, p-values were adjusted based on the Bonferroni method. * indicates a statistically significant difference (adjusted *p*<0.05) vs. CTL.

The unbound maximum plasma concentration (C_max_) of bortezomib after intravenous administration ranges from ~39–98 nM [18]. In the current studies, we determined the effects of bortezomib on OATP1B1- and OATP1B3-mediated transport at various concentrations, including the clinically relevant concentrations (50 and 100 nM). CCK-8 is a specific substrate of OATP1B3, while [^3^H]E217G and [^3^H]pitavastatin are dual substrates of OATP1B1 and OATP1B3 [30]. The effects of bortezomib on OATP1B3-mediated transport in HEK293-OATP1B3 cells were determined using all three substrates, [^3^H]CCK-8, [^3^H]E_2_17βG and [^3^H]pitavastatin, and in human SCH using [^3^H]CCK-8 as the probe substrate. As shown in Fig 5A, co-incubation with positive control rifampicin (25 μM) significantly decreased OATP1B3-mediated [^3^H]CCK-8 accumulation (1 μM, 3 min) to 0.02 ± 0.16 fold of control (Bonferroni-adjusted *p*<0.0001). Without bortezomib pretreatment, bortezomib (10–250 nM) did not affect OATP1B3-mediated [^3^H]CCK-8 accumulation (1 μM, 3 min) (Bonferroni-adjusted *p*-value = 1.0; Fig 5A). However, after pretreatment with bortezomib (50–250 nM, 2 and 7 h), OATP1B3-mediated [^3^H]CCK-8 accumulation was significantly decreased, ranging from 0.65 ± 0.03–0.73 ± 0.02 folds of control (Fig 5B). In human SCH pretreated with bortezomib at 50 and 250 nM for 7 h, accumulation of [^3^H]CCK-8 (1 μM, 3 min) was significantly decreased to 0.64 ± 0.05 and 0.62 ± 0.07 fold of control, respectively (Fig 5C).

The OATP1B3-mediated uptake of [^3^H]pitavastatin (1 μM) was linear, at least up to 90 sec (S4 Fig). A one-minute incubation time period was used for subsequent [^3^H]pitavastatin (1 μM) accumulation assays. Accumulation of OATP1B3-mediated [^3^H]pitavastatin (Fig 5D) and [^3^H]E_2_17βG (Fig 5E) in bortezomib-pretreated cells (50–250 nM, 2 and 7 h) was not significantly different from that in cells pretreated with vehicle control.

We further determined whether the inhibitory effect of bortezomib pretreatment on OATP1B3-mediated CCK-8 accumulation was reversible. Similar to the trend shown in Fig 5B, bortezomib pretreatment (50 nM, 2 h) significantly decreased [^3^H]CCK-8 accumulation to 0.71 ± 0.02 fold of vehicle control pretreatment (Fig 5F, 0 h). At 1, 2, 4 and 6 h after cells were cultured in bortezomib-free media following initial pretreatment with bortezomib (50 nM, 2 h) or vehicle control, [^3^H]CCK-8 accumulation in bortezomib-pretreated cells (Fig 5F grey bars) was still significantly lower than in vehicle control (CTL)-pretreated cells (Fig 5F black bars), which are 0.70 ± 0.03, 0.65 ± 0.02, 0.67 ± 0.03 and 0.70 ± 0.04 fold of CTL, respectively (*p*<0.01 vs. CTL). At 24 h after cells were cultured in bortezomib-free media, there was no significant difference in the accumulation of [^3^H]CCK-8 between bortezomib-pretreated cells compared to CTL (Fig 5F).

### Effects of bortezomib on the transport kinetics of CCK-8 and surface protein levels of OATP1B3 in HEK293-OATP1B3 cells

**Fig 6:**
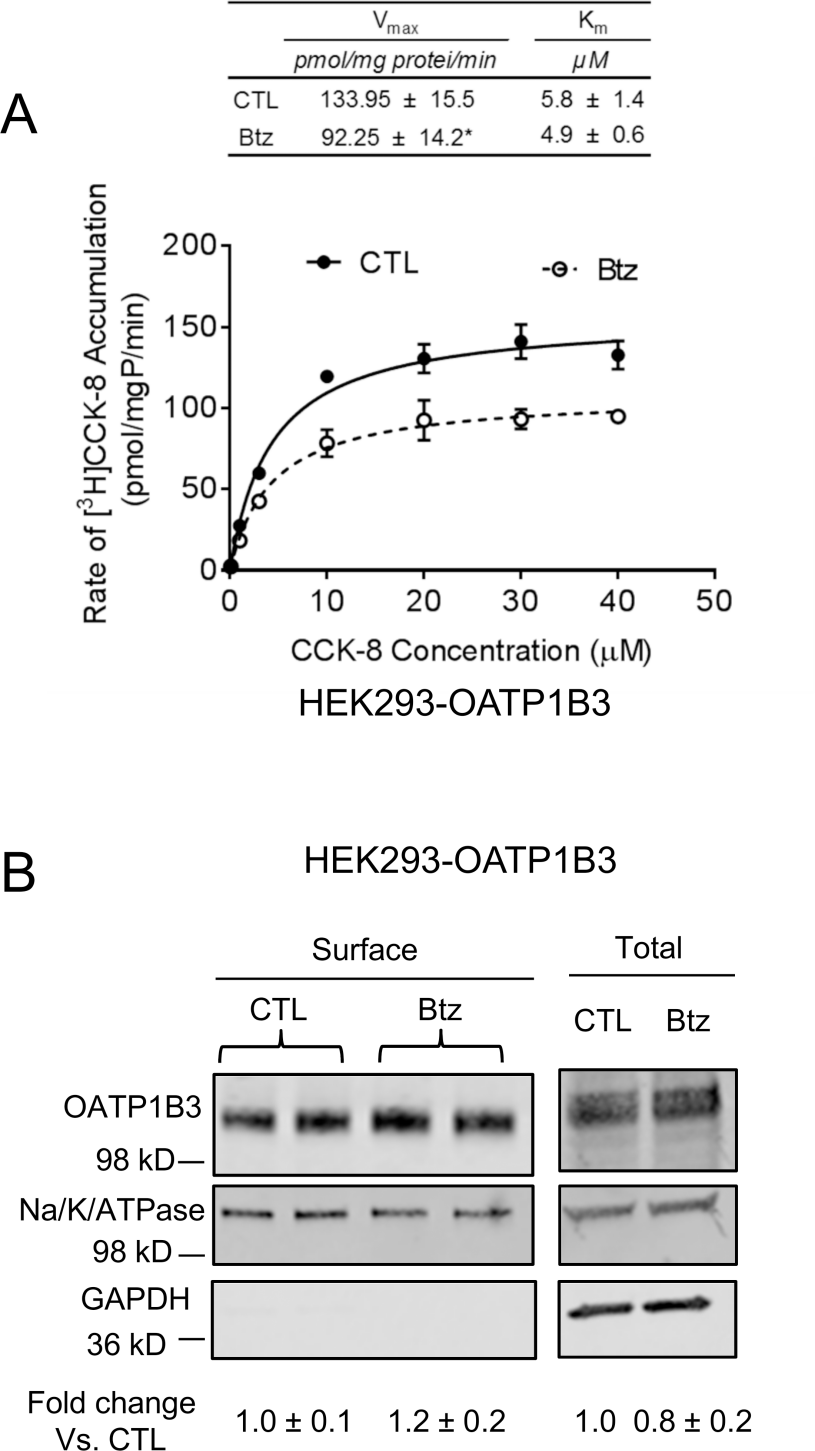
Effects of bortezomib treatment on the transport kinetics of CCK-8 and surface protein levels of OATP1B3 in HEK293-OATP1B3 cells. HEK293-OATP1B3 cells were seeded at a density of 1.2 x 10^5^ cells/well in 24-well plates, and were cultured for 48 h prior to performing the experiment. (A) The concentration-dependent accumulation of CCK-8 (0.1–40 μM, 3 min) was determined in HEK293-OATP1B3 cells pretreated with control (CTL) or bortezomib (Btz) (50 nM, 7 h). Solid and dashed lines represent the best fit lines of the Michaelis–Menten equation to the data of vehicle control (CTL) (closed circles) and bortezomib (Btz) pretreatment (open circles), respectively. A representative graph of three independent experiments performed in triplicate is shown. The student’s t-test was conducted to compare the V_max_ and K_m_ values between bortezomib and vehicle control pretreatment. * indicates a statistically significant difference (*p*<0.05; bortezomib vs. CTL). (B) HEK293-OATP1B3 cells were pretreated with 50 nM bortezomib (Btz) or vehicle control (CTL) for 7 h. Surface levels of OATP1B3 were determined via biotinylation, followed by immunoblotting with OATP1B3 and Na-K-ATPase antibodies. GAPDH was used as a cytoplasmic protein marker. OATP1B3 surface protein levels were determined by densitometry and were normalized to those of Na-K-ATPase. Fold changes in the surface levels of OATP1B3 (bortezomib vs. CTL) were expressed as mean ± SD of three independent experiments.

HEK293-OATP1B3 cells were pretreated with bortezomib (50 nM) or vehicle control for 7 h. As shown in Fig 6A, bortezomib pretreatment (50 nM, 7 h) significantly decreased the maximal transport velocity (V_max_) values of CCK-8 transport compared with vehicle control treatment (92.25 ± 14.2 vs. 133.95 ± 15.5 pmol/mg protein/min, *p* < 0.05), without affecting the K_m_ values (4.9 ± 0.6 vs. 5.8 ± 1.4 μM) (mean ± SD, n = 3).

The surface biotinylation assay was conducted to determine the effects of bortezomib pretreatment (50 nM, 7 h) on the cell surface protein levels of OATP1B3 in HEK293-OATP1B3 cells. As shown in Fig 6B, OATP1B3 surface protein levels in bortezomib-treated cells after normalization with Na-K-ATPase were 1.2 ± 0.2 fold of control (Mean ± SD, n = 3). The total protein levels of OATP1B3 in bortezomib-treated cells were 0.82 ± 0.2 fold of control (Mean ± SD, *n* = 3). GAPDH was detected only in the whole cell lysates, but not in the cell surface fraction, suggesting that the surface protein fraction was not contaminated with intracellular protein.

### Effects of bortezomib on OATP1B1-mediated [^3^H]pitavastatin and [^3^H]E_2_17βG transport in HEK293-OATP1B1 cells and on [^3^H]pitavastatin accumulation in human SCH

**Fig 7:**
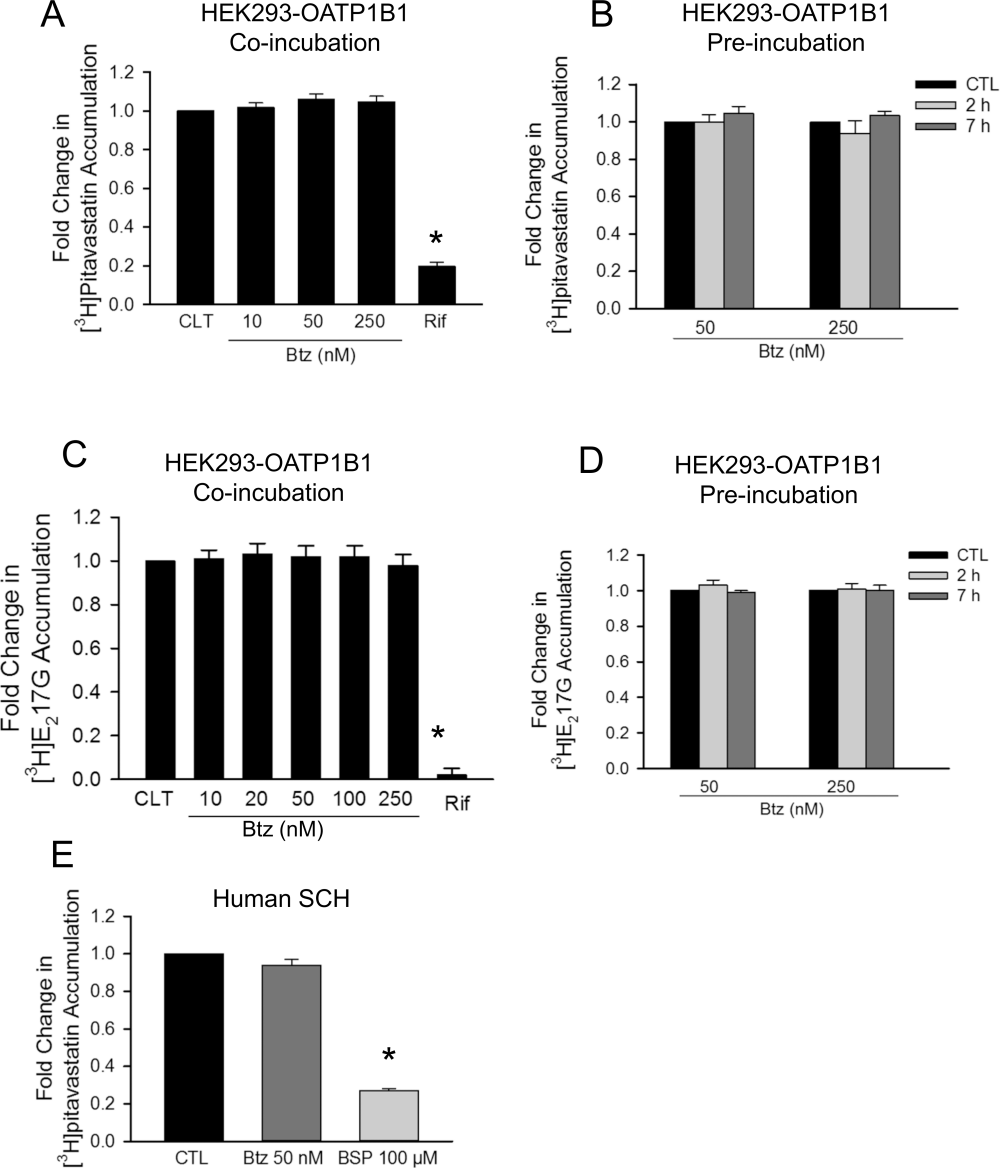
Effects of bortezomib on OATP1B1-mediated [^3^H]pitavastatin and [^3^H]E_2_17βG transport in HEK293-OATP1B1 cells and on [^3^H]pitavastatin accumulation in human SCH. HEK293-OATP1B1 cells were seeded at a density of 1.2 x 10^5^ cells/well in a 24-well plate and were cultured to confluence. Human SCH were cultured as described in the “Materials and Methods”. (A) Model-estimated fold change and associated SE in [^3^H]pitavastatin accumulation (1 μM, 0.5 min) in the presence of 10–250 nM bortezomib (Btz) or 25 μM rifampicin (Rif) vs. vehicle control (CTL) in HEK293-OATP1B1 cells (Co-incubation). (B) Model-estimated fold change and associated SE in [^3^H]pitavastatin accumulation (1 μM, 0.5 min) in HEK293-OATP1B1 cells pretreated with bortezomib (Btz) vs. vehicle control (CTL) at each indicated time and concentration (Pre-incubation). Following pretreatment, cells were washed three times with HBSS buffer, and the [^3^H]pitavastatin accumulation was determined in the absence of bortezomib. (C) Model-estimated fold change and associated SE in [^3^H]E_2_17βG accumulation in the presence of 10–250 nM bortezomib (Btz) or 25 μM rifampicin (Rif) vs. vehicle CTL in HEK293-OATP1B1 cells (Co-incubation). (D) Model-estimated fold change and associated SE in [^3^H]E_2_17βG accumulation (1 μM, 2 min) vs. vehicle CTL treatment in HEK293-OATP1B1 cells pretreated with bortezomib (Btz) for the indicated times and at the indicated concentrations (Pre-incubation). Following pretreatment, cells were washed three times with HBSS buffer, and the [^3^H]E_2_17βG accumulation was determined in the absence of bortezomib. (E) Model-estimated fold change and associated SE of [^3^H]pitavastatin accumulation (1 μM, 0.5 min) in human SCH pre-incubated with bortezomib (50 nM for 7 h) or co-incubated with positive control bromosulfophthalein (BSP) (100 μM) vs. vehicle CTL. Fold changes and SEs were estimated by linear mixed effects models, as described in the “Data Analysis” section (n = 3 hepatocyte donors in triplicate). To account for multiple comparisons, p-values were adjusted based on the Bonferroni method. * indicates a statistically significant difference (adjusted *p*<0.05) vs. CTL.

Pitavastatin and E_2_17βG, which are sensitive substrates used to study the inhibitory effects toward OATP1B1 [29, 42], were used in the current studies as probe substrates to determine the effects of bortezomib on OATP1B1-mediated transport in HEK293-OATP1B1 cells. [^3^H]pitavastatin was also used as a clinically relevant OATP drug substrate in human SCH [10, 29]. In HEK293-OATP1B1 cells, co-incubation with the positive control rifampicin (25 μM), but not bortezomib (up to 250 nM), significantly decreased the accumulation of [^3^H]pitavastatin (1 μM, 0.5 min) (Fig 7A) and [^3^H]E_2_17βG (1 μM, 2 min) (Fig 7C). Pretreatment with bortezomib (50 and 250 nM, 2 and 7 h) did not significantly affect the accumulation of [^3^H]pitavastatin (1 μM, 0.5 min) (Fig 7B) or [^3^H]E_2_17βG (1 μM, 2 min) (Fig 7D). In human SCH, co-incubation with positive control bromosulfophthalein (BSP) (100 μM) significantly decreased [^3^H]pitavastatin accumulation to 0.27 ± 0.01 fold of control (Fig 7E), while pretreatment with bortezomib (50 nM) (Fig 7E) did not significantly affect [^3^H]pitavastatin accumulation (1 μM, 0.5 min).

### Effects of proteasome inhibitors MG132, epoxomicin and carfilzomib on OATP1B1- and 1B3-mediated transport

**Fig 8:**
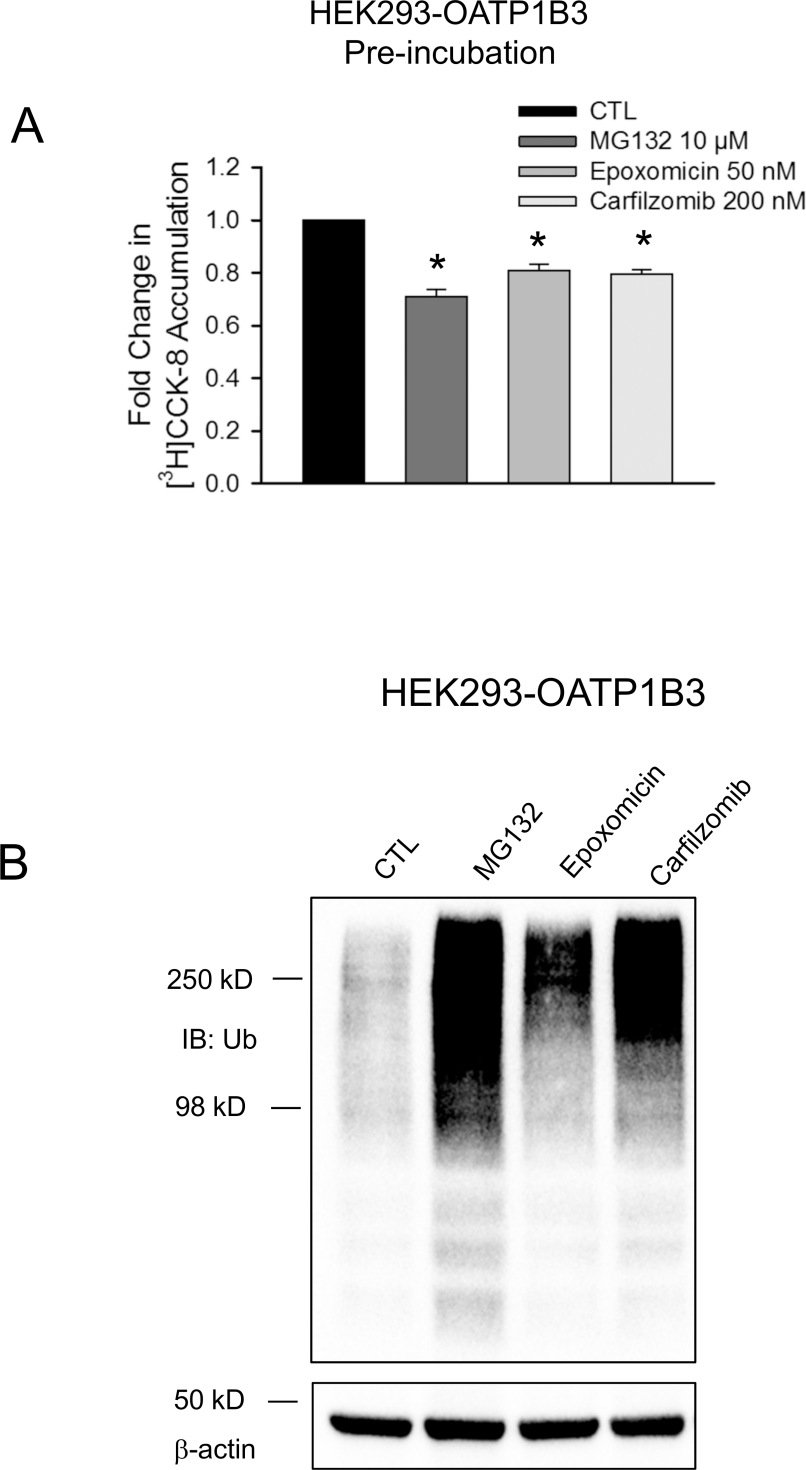
Effects of proteasome inhibitors MG132, epoxomicin and carfilzomib on OATP1B3-mediated transport and total ubiquitin-conjugated proteins in HEK293 stable cell lines. HEK293-OATP1B3 cells were seeded at a density of 1.2 x 10^5^ cells/well in 24-well plates and cultured to confluence. (A) Model-estimated fold change and associated SE in [^3^H]CCK-8 accumulation (1 μM, 3 min) in HEK293-OATP1B3 cells pretreated with MG132 (10 μM), epoxomicin (50 nM) and carfilzomib (200 nM) for 2 h vs. vehicle control (CTL) pretreatment. Fold changes and SE were estimated by linear mixed effects models, as described in the “Data Analysis” section (n = 3 in triplicate). To account for multiple comparisons, p-values were adjusted based on the Bonferroni method. * indicates a statistically significant difference (adjusted p<0.05) vs. CTL. (B) Immunoblot of ubiquitin was conducted in whole cell lysates of HEK293-OATP1B3 cells pretreated with MG132 (10 μM), epoxomicin (50 nM) and carfilzomib (200 nM) or vehicle control for two hours. β-actin served as the loading control for the whole cell lysates.

The effects of pretreatment with other proteasome inhibitors, MG132, epoxomicin and carfilzomib, on OATP1B3- and OATP1B1-mediated transport were determined in HEK293-OATP1B3 and HEK293-OATP1B1 cells, respectively. Carfilzomib is a second-generation proteasome inhibitor drug with an unbound C_max_ of 176 nM [43]. A clinically relevant concentration of 200 nM was used for carfilzomib treatment in the current studies. Pretreatment with MG132 (10 μM), epoxomicin (50 nM) or carfilzomib (200 nM) for 2 h significantly decreased [^3^H]CCK-8 accumulation to 0.71 ± 0.01, 0.81 ± 0.04, and 0.79 ± 0.02 fold of control, respectively (p < 0.05 vs. control) (Fig 8A). The efficient inhibition of proteasome activity by current treatment condition was confirmed by immunoblot with ubiquitin. Total ubiquitin-conjugated proteins were markedly increased in HEK293-OATP1B3 cells treated with proteasome inhibitors (Fig 8B), consistent with the efficient blocking of protein degradation via the ubiquitin proteasome system, similar to what was observed previously. Pretreatment with MG132 (10 μM), epoxomicin (50 nM) and carfilzomib (200 nM) for 2 h did not significantly affect OATP1B1-mediated transport of [^3^H]E_2_17βG (1 μM, 2 min) in HEK293-OATP1B1 cells (S5 Fig).

### Lactate dehydrogenase (LDH) cytotoxicity

LDH assay revealed negligible cytotoxicity in HEK293 stable cell lines and in human SCH treated with bortezomib (250 nM, 7 h) (S6 Fig).

## Discussion

OATP1B1 and OATP1B3 mediate the hepatic uptake of a diverse array of clinically important drugs; therefore, understanding the regulation of OATP1B1 and OATP1B3 transport function, especially by commonly administered therapeutic drugs, has significant clinical relevance in predicting potential OATP-mediated DDIs. In the current studies, we determined the ubiquitin modification of OATP1B1 and OATP1B3 and the effects of proteasome inhibitors on OATP1B1 and OATP1B3-mediated transport.

In order to determine the ubiquitination of OATP1B1 and OATP1B3, we first examined whether OATP1B1 and OATP1B3 can be conjugated with HA-ubiquitin. As shown in Fig 1 (lanes 1 and 9 of A and B), the HA-Ub-conjugated FLAG-tagged OATP1B1 and OATP1B3 proteins were only observed in cells in which HA-Ub was co-expressed with FLAG-OATP1B1/OATP1B3. These results demonstrate, for the first time, that OATP1B1 and OATP1B3 can be ubiquitin-modified.

FLAG immunoblots in non-boiled whole cell lysates detected FLAG-OATP1B1 and FLAG-OATP1B3 at major molecular weights of ~98 kD and ~130 kD, respectively (lanes 18 of Fig 1A and 1B). These molecular weights are comparable to previously published molecular weights of OATP1B1 and OATP1B3 in HEK293-OATP1B1 and HEK293-OATP1B3 cells, respectively [26, 27, 29]. The HA-ubiquitin-conjugated FLAG-OATP1B1 and FLAG-OATP1B3 proteins (lanes 1 and 9 of Fig 1A and 1B) appear to have molecular weights that are higher than the regular molecular weight of FLAG-OATP1B1 (~98 kD) and FLAG-OATP1B3 (~130 kD). Ubiquitination often increases the molecular weight of target proteins, as has been reported for other proteins [14, 44]. The higher than regular molecular weights of the HA-ubiquitin-conjugated FLAG-OATP1B1 and FLAG-OATP1B3 proteins (lanes 1 and 9 of Fig 1A and 1B) may attributed to the ubiquitin-modification of the transporter proteins.

Higher molecular weight bands (~250 kD) of FLAG-OATP1B1 and FLAG-OATP1B3 were observed in FLAG immunoblotting after FLAG immunoprecipitation in cells transiently expressing FLAG-OATP1B1 and/or FLAG–OATP1B3 (Fig 1A and 1B, lanes 13 and 15). Several factors may be involved in the appearance of the higher molecular weight bands of FLAG-OATP1B1 and FLAG-OATP1B3 mentioned above. First, after immunoprecipitation, the immunocomplexes were eluted from the protein A/G resin by boiling, per a routine method according to the manufacturer’s instructions. It has been well documented that membrane proteins tend to form higher molecular weight aggregates when boiled [45, 46]. We observed a similar phenomenon for FLAG-OATP1B1 and FLAG-OATP1B3. As shown in the supplemental results (S7 Fig), the higher molecular weight bands of FLAG-OATP1B1 and FLAG-OATP1B3 (~250 kD) from the whole cell lysate samples were prominent after boiling, while these bands were minimal without boiling. Recently, OATP1B3 was reported to be able to form homo-oligomers [47]. In this report, in the absence of crosslinking agent, OATP1B3 eluted from immunoprecipitation without boiling had a major molecular weight of >100 kD (monomer), while in the presence of crosslinking agent, homo-oligomer OATP1B3 was detected at higher molecular weight of >150 kD [47]. This report [47], together with the results shown in S7 Fig, suggest that the observed higher molecular weight FLAG-OATP1B1 and FLAG-OATP1B3 bands (~250 kD) in the immunoprecipitation samples (Fig 1A and 1B, lanes 13 and 15) might be related to protein aggregation or oligomerization after boiling [45, 46]. Second, in a previous report [48], treatment with tunicamycin, an inhibitor of protein N-linked glycosylation, markedly reduced the amount of high molecular weight band of OATP1B1. Thus, post-translational modification, such as glycosylation, may also be involved in the formation of the high molecular weight bands of OATP1B1 and OATP1B3 observed in current studies (Fig 1A and 1B, lanes 13 and 15).

The ubiquitin proteasome system is not only involved in the degradation of cytosolic proteins [49], but also in the degradation of some plasma membrane proteins, including transport proteins, such as P-gp [12], sodium taurocholate co-transporting polypeptide (NTCP), [13] and apical sodium-dependent bile acid transporter (ASBT) [14]. Based on the finding that OATP1B1 and OATP1B3 can be conjugated to HA-ubiquitin (Fig 1), we further assessed the potential involvement of the ubiquitin-proteasome system in degradation of OATP1B1 and OATP1B3. Bortezomib treatment (50 nM, 7 h) in all conditions reported in the current manuscript efficiently blocked proteasome activity, as indicated by a marked increase in total ubiquitin-conjugated proteins after bortezomib treatment (Fig 2A and 2B right panels, S2E and S2F Fig). After bortezomib treatment, there was an increase in the amount of endogenous ubiquitin-conjugated-FLAG-OATP1B1 and -FLAG-OATP1B3 (Fig 2A and 2B left panels). However, there was no obvious increase in the total protein levels of OATP1B1 and OATP1B3 in transporter-overexpressing HEK293 cells, either at the regular molecular weight or at the higher molecular weight position due to potential ubiquitination (Fig 2A and 2B middle panels). Bortezomib treatment did not affect the total protein levels of OATP1B1 and OATP1B3 in human SCH (Fig 3A) or in HEK293 stable cell lines expressing untagged OATP1B1 and OATP1B3 (S2A–S2D Fig). These data support that the ubiquitin-proteasome may not be the major degradation pathway of OATP1B1 and OATP1B3 under the current conditions. Previously, we reported that the lysosome is involved in the degradation of OATP1B1 [10]. In current studies, we found that treatment with the lysosome inhibitor chloroquine markedly increased the total protein levels of OATP1B3 not only in the HEK293-OATP1B3 stable cell line (Fig 4A), but also in human SCH (Fig 4B), suggesting that the lysosome is also involved in the degradation of OATP1B3. Taken together, our data suggest that compared with the lysosome pathway, the ubiquitin-proteasome system may play a less important role in the degradation of OATP1B1 and OATP1B3, at least under the current conditions. The ubiquitination of OATP1B1 and OATP1B3 were also determined in transporter-overexpressing HEK293 cells (Figs 1 and 2). The ubiquitination of endogenous OATP1B1 and OATP1B3 and the effects of proteasome inhibitors on the ubiquitination of endogenous OATP1B1 and OATP1B3 warrant further characterization in primary human hepatocytes using validated OATP1B1 and OATP1B3 antibodies that can specifically immunoprecipitate OATP1B1 and OATP1B3, respectively.

Treatment with proteasome inhibitors has been associated with the altered function of transport proteins. For example, proteasome inhibition decreases serotonin transporter (SERT)-mediated serotonin uptake [16] and the efflux function of P-glycoprotein (P-gp) [17]. In the current studies, we determined the effects of proteasome inhibitors on OATP1B1- and OATP1B3-mediated transport. Bortezomib is not a competitive inhibitor of OATP1B1 and OATP1B3, at least at concentrations up to 250 nM (Fig 5A and Fig 7A and 7C). However, pretreatment with bortezomib (50–250 nM, 2–7 h) significantly decreases OATP1B3-mediated [^3^H]CCK-8 accumulation in HEK293-OATP1B3 stable cell line and in human SCH (Fig 5B and 5C). The decrease in OATP1B3-mediated CCK-8 transport induced by bortezomib pretreatment (50 nM, 2 h) lasted for at least 6 h after culturing in bortezomib-free media (Fig 5F), however, was no longer evident after culturing the cells for 24 h in bortezomib-free media (Fig 5F). Treatment with other proteasome inhibitors MG132, epoxomicin and carfilzomib also significantly decreased OATP1B3-mediated CCK-8 transport (Fig 8A). OATP1B1 and OATP1B3 share a variety of common substrates, such as statins and estradiol-17-β-glucuronide [7, 30]. However, some substrates, such as CCK-8, tend to be transported specifically by OATP1B3 [8, 30, 50]. Interestingly, the inhibitory effects of bortezomib pretreatment appear to be specific to OATP1B3-mediated CCK-8 transport (Fig 5B and 5C), as bortezomib pretreatment did not affect transport of [^3^H]E_2_17βG and [^3^H]pitavastatin mediated by either OATP1B3 (Fig 5D and 5E) or OATP1B1 (Fig 7B and 7D). Although pitavastatin is a substrate of multiple hepatic OATPs, including OATP1B1 and OATP1B3, OATP1B1 plays a primary role in hepatic uptake of pitavastatin [5]. The lack of inhibitory effects of bortezomib pretreatment on pitavastatin accumulation mediated by OATP1B1 (Fig 7B) and OATP1B3 (Fig 5D) is consistent with the finding that pretreatment with bortezomib did not affect pitavastatin accumulation in human SCH (Fig 7E). Currently, the mechanism(s) through which bortezomib pretreatment reduces OATP1B3-mediated transport in a substrate-dependent manner remains unknown. Considering such pretreatment effects are associated with the OATP1B3-specific substrate CCK-8, but not with other dual substrates of OATP1B1 and OATP1B3, such as pitavastatin and E_2_17βG, we speculate that bortezomib pretreatment may affect conformation or binding sites of OATP1B3 that are specifically associated with CCK-8 transport.

In an effort to elucidate the potential mechanisms underlying the decreased OATP1B3-mediated transport of [^3^H]CCK-8 after bortezomib treatment, we determined the effects of bortezomib on the OATP1B3-mediated transport kinetics of CCK-8. Bortezomib pretreatment (50 nM, 7 h) markedly decreased the V_max_ values of OATP1B3-mediated CCK-8 transport without apparently affecting the K_m_ values of CCK-8 transport (Fig 6A), suggesting that bortezomib pretreatment mainly affects the capacity of OATP1B3-mediated transport but not the substrate affinity of the transporter. The decreased V_max_ values of transport proteins after pretreatment have been reported to be associated with reduced membrane levels of the transporter proteins in some studies [51, 52], while likely associated with a decrease in the turn-over rate of the transporter, but not altered surface levels of the transporter protein [53]. In the current studies, no obvious changes in the surface levels of OATP1B3 were observed after bortezomib treatment (50 nM, 7 h) (Fig 6B), suggesting that the decreased V_max_ of OATP1B3-mediated CCK-8 transport after bortezomib treatment is not associated with apparent changes in surface levels of OATP1B3. The exact mechanism underlying the bortezomib pretreatment effect on OATP1B3-mediated CCK-8 transport warrants further characterization in the future.

In conclusion, the present studies for the first time report that OATP1B1 and OATP1B3 can be ubiquitin-modified. Pretreatment with bortezomib results in moderate reduction of OATP1B3-mediated transport in a substrate-dependent manner, while it does not affect OATP1B1-mediated transport. Taken together, our data support that bortezomib has low potential to cause OATP-mediated clinical DDIs.

## Supporting information

S1 Fig Characterization of HEK293-FLAG-OATP1B3 stable cell line. Immunoblot in HEK293-FLAG-OATP1B3 cells with OATP1B3 (A) and FLAG (B) antibody. β-actin served as the loading control. (C) Accumulation of [^3^H]CCK-8 (1 μM, 3 min) in HEK293-FLAG-OATP1B3 and Mock cells.
(TIF)

S2 Fig Effects of bortezomib on total protein levels of OATP1B1 and OATP1B3 and on total ubiquitin-conjugated proteins in HEK293-OATP1B1 and–OATP1B3 stable cell lines. HEK293-OATP1B1 and -OATP1B3 cells were seeded in 24-well plates at a density of 1.2 x 10^5^ cells/well, and were cultured for 48 h prior to treatment with bortezomib (btz) at the indicated concentration and time duration. Immunoblot of OATP1B1 (A and C), OATP1B3 (B and D) and ubiquitin (E and F) were conducted in whole cell lysates from HEK293-OATP1B1 or HEK293-OATP1B3 cells. β-actin served as the loading control. In A-D, OATP1B1 and OATP1B3 protein levels determined by densitometry were normalized to levels of β-actin. Fold changes of total protein levels in bortezomib-treated cells vs. CTL were expressed as mean ± SD (n = 3). Representative immunoblot images are shown from at least 3 independent experiments.
(TIF)

S3 Fig Immunoblot of OATP1B1 and OATP1B3 in human SCH. Human SCH were treated with bortezomib or vehicle control for 7 h at 50 or 250 nM as indicated in the figure legend. **(A)** immunoblot of OATP1B1 (left panel) and OATP1B3 (right panel) in whole cell lysates of human SCH (donor GC 4008), HEK293-Mock and HEK293-OATP1B3 stable cell lines. The blot was first probed with an OATP1B1 antibody (left panel). After stripping, the same blot was reprobed with an OATP1B3 antibody (right panel). The bracket denotes the non-specific bands detected by the OATP1B1 antibody in HEK293-Mock and HEK293-OATP1B3 cells. Arrows indicate the specific bands of OATP1B1 and OATP1B3. **(B)** Immunoblot of OATP1B3 and β-actin in whole cell lysates of human SCH (donor HUM 4130). Blot was first probed with the OATP1B3 antibody (left panel), and subsequently probed with β-actin antibody without stripping.
Note: * in the OATP1B3 immunoblot (A right panel) denotes the residual signal coming from the OATP1B1 immunoblot that was not completely removed after stripping. These * denoted bands are superimposable with the bands in the OATP1B1 blot (A left panel). In B, when a naïve blot was first probed with OATP1B3 antibody, only one specific OATP1B3 band is detected.
(TIF)

S4 Fig Time-dependent accumulation of [^3^H]pitavastatin in HEK293-OATP1B3 and HEK293-Mock cells. Time-dependent accumulation of [^3^H]pitavastatin (1 μM) was determined in HEK293-OATP1B3 (open circle) and HEK293-Mock cells (open square) at the indicated time points. The OATP1B3-mediated accumulation of [^3^H]pitavastatin (1 μM) (closed circle) was determined as the difference between the accumulation of [^3^H]pitavastatin in HEK293-OATP1B3 and that in HEK293-Mock cells. Data represent mean ± SD (n = 1 in triplicate).
(TIF)

S5 Fig Effect of proteasome inhibitors MG132, epoxomicin, and carfilzomib on OATP1B1-mediated [^3^H] E_2_17G transport in HEK293-OATP1B1 cells. HEK293-OATP1B1 cells were seeded at 1.2 x 10^5^ cells/well in a 24-well plate and cultured for 48 h. Model-estimated fold change and associated SE in [^3^H]E_2_17G accumulation vs. CTL in HEK293-OATP1B1 cells pretreated for 2 h with MG132 (10 μM) or epoxomicin (50 nM) or carfilzomib (200 nM). Fold changes and SE were estimated by linear mixed effects models, as described in the Data Analysis section (n = 3 in triplicate). To account for multiple comparisons, p-values were adjusted based on the Bonferroni method.
(TIF)

S6 Fig Cytotoxicity in HEK293-OATP1B1, -OATP1B3, -FLAG-OATP1B1, -FLAG-OATP1B3, and human SCH treated with bortezomib. An LDH assay was performed to measure the cytotoxicity in HEK293 stable cell lines expressing OATP1B1, OATP1B3, FLAG-OATP1B1 or FLAG-OATP1B3, and human SCH treated for 7 h with 250 nM bortezomib. Triton-X (2%)-treated cells and media-treated cells served as the 100% cytotoxicity positive control and negative control, respectively. Data represents mean ± SD (n = 1 in triplicate).
(TIF)

S7 Fig FLAG immunoblot. The HEK293 cells were transiently transfected with pCMV6-FLAG-OATP1B1 (A) and pCMV6-FLAG-OATP1B3 (B). Forty-eight hours after transfection, whole cell lysates were immunoblotted with FLAG antibody, with and without boiling at 100°C for 5 min. Representative images from n = 2 are shown.
(TIF)

## Abbreviations

ABC: ATP-binding cassette
E_2_17βG: estradiol 17β-D-glucuronide
BSA: bovine serum albumin
DDIs: drug-drug interactions
DMEM: Dulbecco's Modified Eagle Medium
DPBS: Dulbecco's Phosphate-Buffered Saline
HBSS: Hanks' Balanced Salt Solution
MEM: minimum essential medium
NEAA: nonessential amino acids
HEK293: human embryonic kidney 293
LDH: lactate dehydrogenase
OATP: organic anion transporting polypeptide
OATs: organic anion transporters
SLCO: solute carrier organic anion
