# Supplementary figures and images for: Treatment with proteasome inhibitor bortezomib decreases organic anion transporting polypeptide (OATP) 1B3-mediated transport in a substrate-dependent manner
Source: PLoS One. 2017 Nov 6;12(11):e0186924. doi: 10.1371/journal.pone.0186924 (PMC5673231; doi:10.1371/journal.pone.0186924)

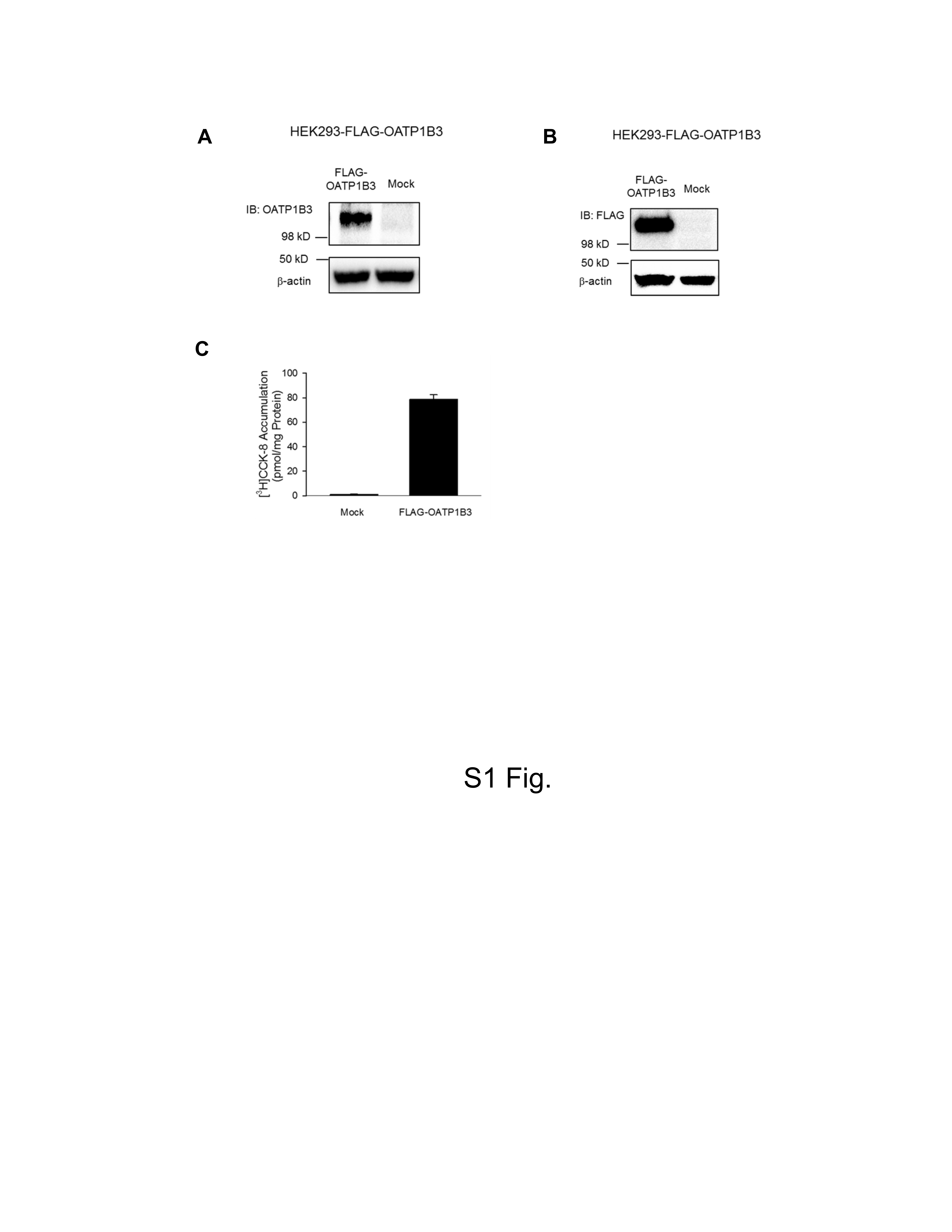

Supplement: S1 Fig — Immunoblot in HEK293-FLAG-OATP1B3 cells with OATP1B3 (A) and FLAG (B) antibody. β-actin served as the loading control. (C) Accumulation of [3H]CCK-8 (1 μM, 3 min) in HEK293-FLAG-OATP1B3 and Mock cells. (TIF) [file pone.0186924.s001.tif]

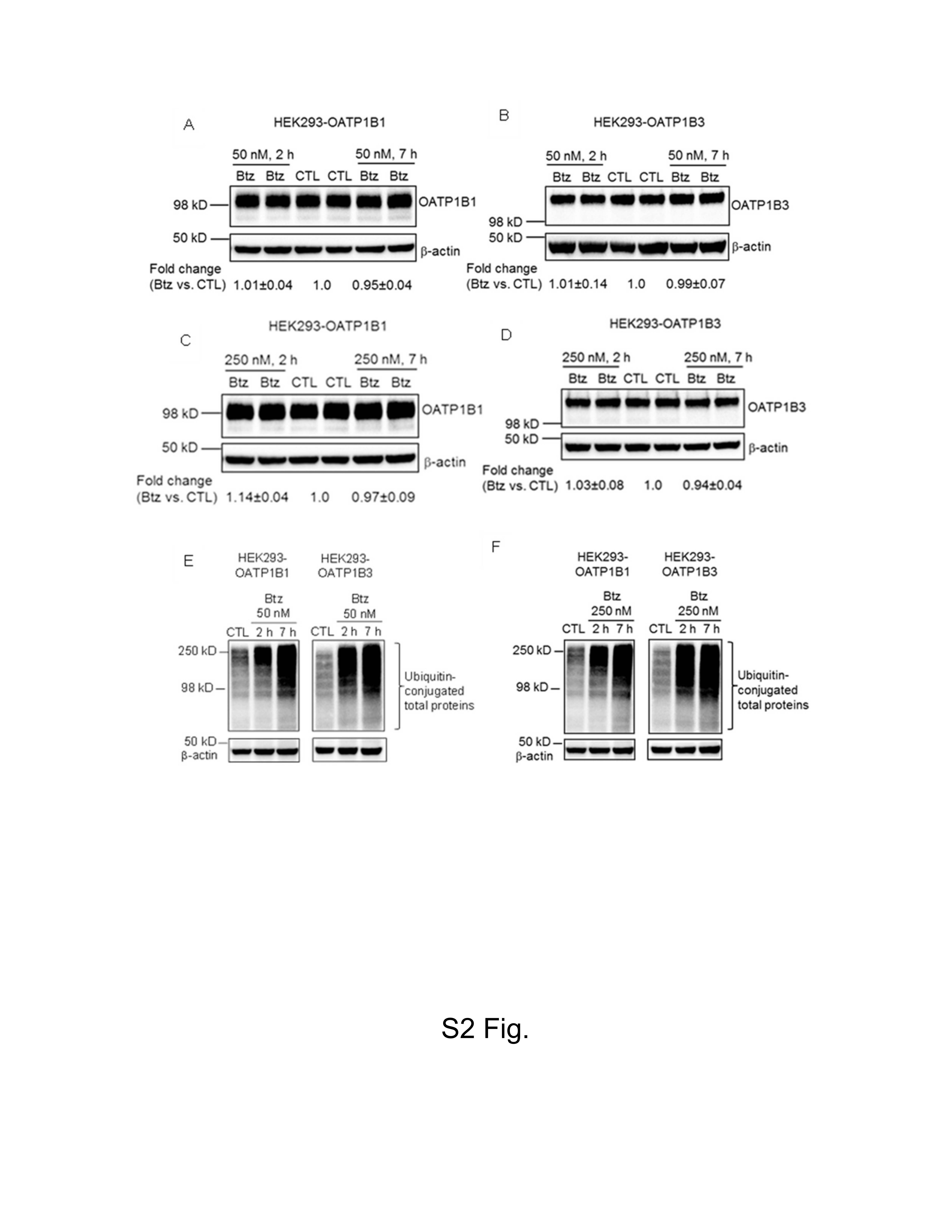

Supplement: S2 Fig — HEK293-OATP1B1 and -OATP1B3 cells were seeded in 24-well plates at a density of 1.2 x 10^5^ cells/well, and were cultured for 48 h prior to treatment with bortezomib (btz) at the indicated concentration and time duration. Immunoblot of OATP1B1 (A and C), OATP1B3 (B and D) and ubiquitin (E and F) were conducted in whole cell lysates from HEK293-OATP1B1 or HEK293-OATP1B3 cells. β-actin served as the loading control. In A-D, OATP1B1 and OATP1B3 protein levels determined by densitometry were normalized to levels of β-actin. Fold changes of total protein levels in bortezomib-treated cells vs. CTL were expressed as mean ± SD (n = 3). Representative immunoblot images are shown from at least 3 independent experiments. (TIF) [file pone.0186924.s002.tif]

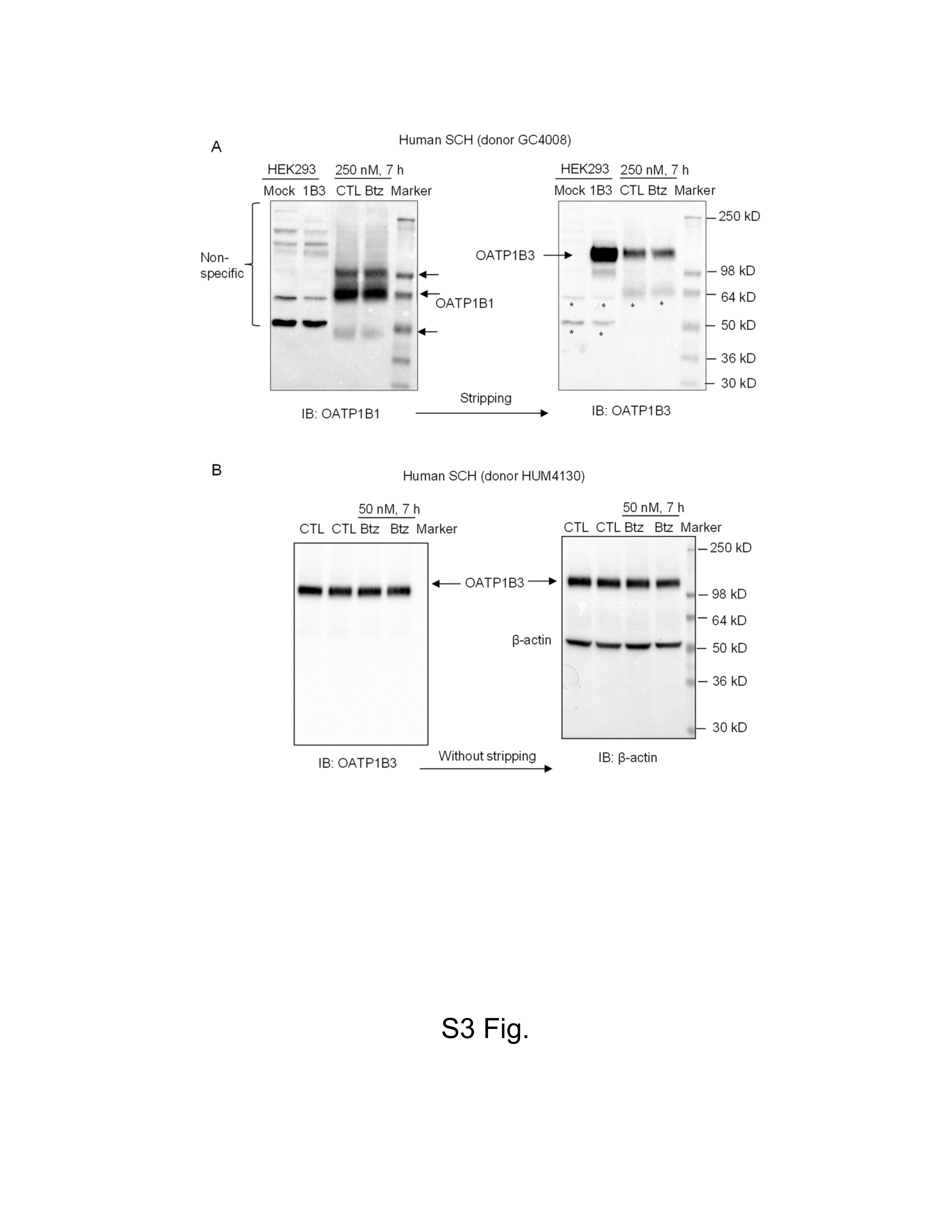

Supplement: S3 Fig — Human SCH were treated with bortezomib or vehicle control for 7 h at 50 or 250 nM as indicated in the figure legend. (A) immunoblot of OATP1B1 (left panel) and OATP1B3 (right panel) in whole cell lysates of human SCH (donor GC 4008), HEK293-Mock and HEK293-OATP1B3 stable cell lines. The blot was first probed with an OATP1B1 antibody (left panel). After stripping, the same blot was reprobed with an OATP1B3 antibody (right panel). The bracket denotes the non-specific bands detected by the OATP1B1 antibody in HEK293-Mock and HEK293-OATP1B3 cells. Arrows indicate the specific bands of OATP1B1 and OATP1B3. (B) Immunoblot of OATP1B3 and β-actin in whole cell lysates of human SCH (donor HUM 4130). Blot was first probed with the OATP1B3 antibody (left panel), and subsequently probed with β-actin antibody without stripping. Note: * in the OATP1B3 immunoblot (A right panel) denotes the residual signal coming from the OATP1B1 immunoblot that was not completely removed after stripping. These * denoted bands are superimposable with the bands in the OATP1B1 blot (A left panel). In B, when a naïve blot was first probed with OATP1B3 antibody, only one specific OATP1B3 band is detected. (TIF) [file pone.0186924.s003.tif]

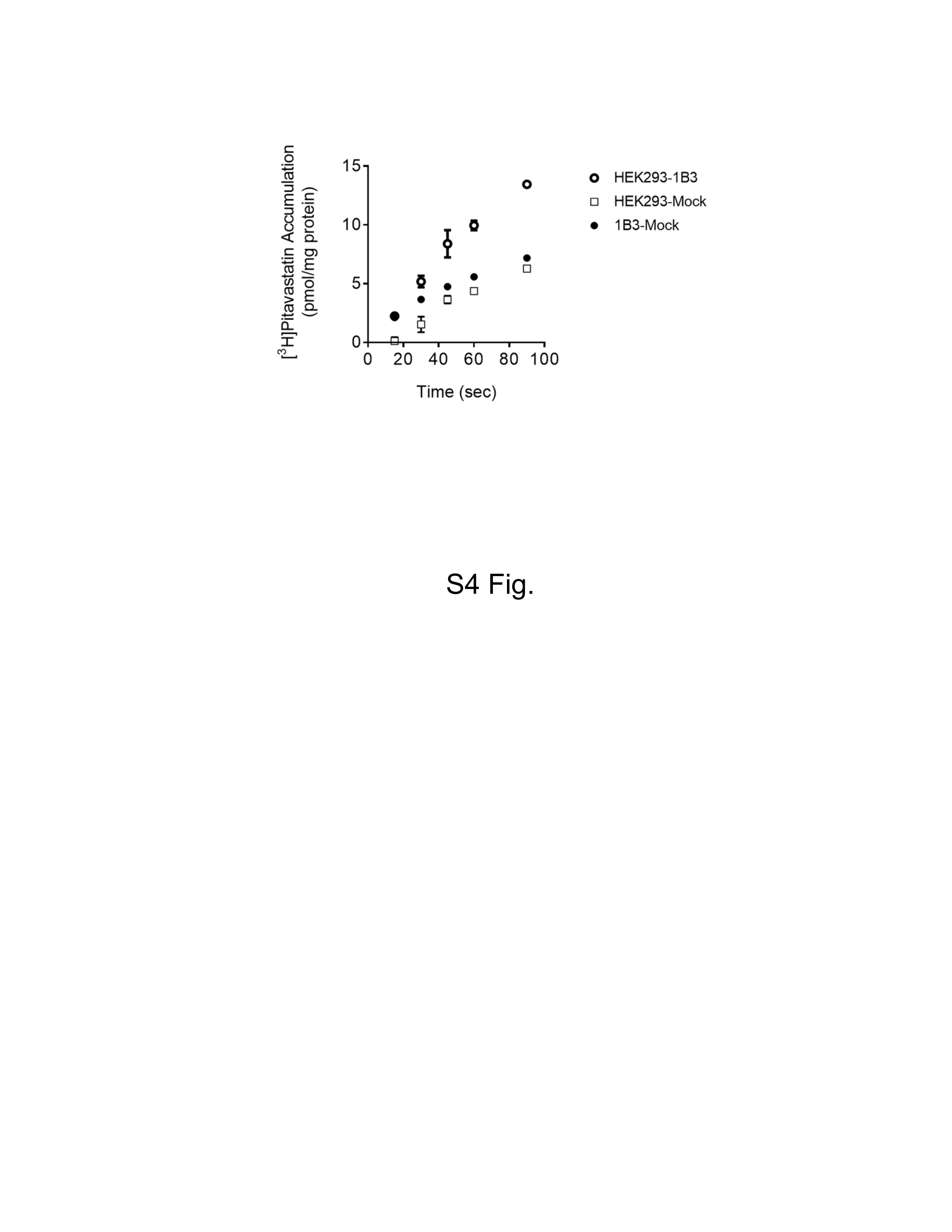

Supplement: S4 Fig — Time-dependent accumulation of [3H]pitavastatin (1 μM) was determined in HEK293-OATP1B3 (open circle) and HEK293-Mock cells (open square) at the indicated time points. The OATP1B3-mediated accumulation of [3H]pitavastatin (1 μM) (closed circle) was determined as the difference between the accumulation of [3H]pitavastatin in HEK293-OATP1B3 and that in HEK293-Mock cells. Data represent mean ± SD (n = 1 in triplicate). (TIF) [file pone.0186924.s004.tif]

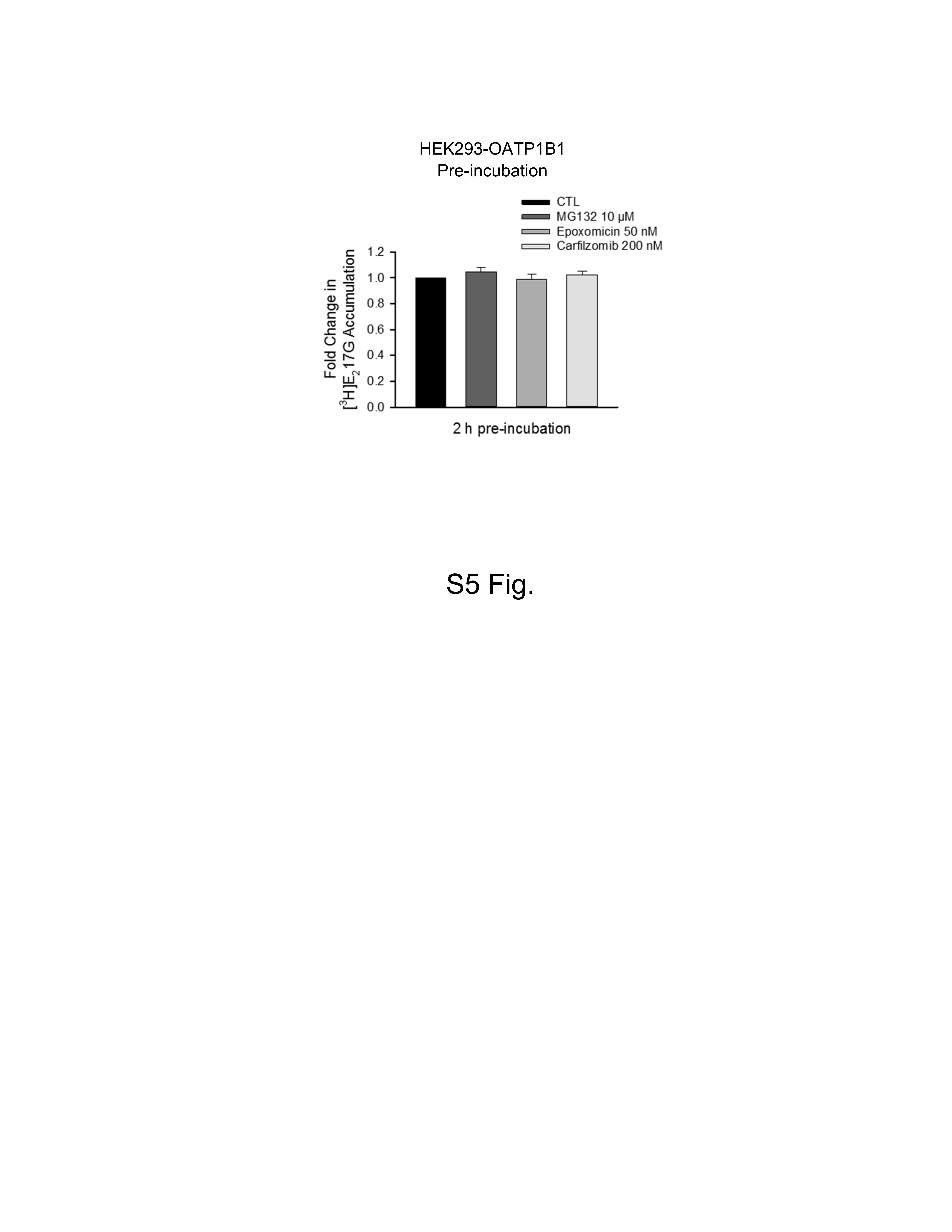

Supplement: S5 Fig — HEK293-OATP1B1 cells were seeded at 1.2 x 10^5^ cells/well in a 24-well plate and cultured for 48 h. Model-estimated fold change and associated SE in [3H]E217G accumulation vs. CTL in HEK293-OATP1B1 cells pretreated for 2 h with MG132 (10 μM) or epoxomicin (50 nM) or carfilzomib (200 nM). Fold changes and SE were estimated by linear mixed effects models, as described in the Data Analysis section (n = 3 in triplicate). To account for multiple comparisons, p-values were adjusted based on the Bonferroni method. (TIF) [file pone.0186924.s005.tif]

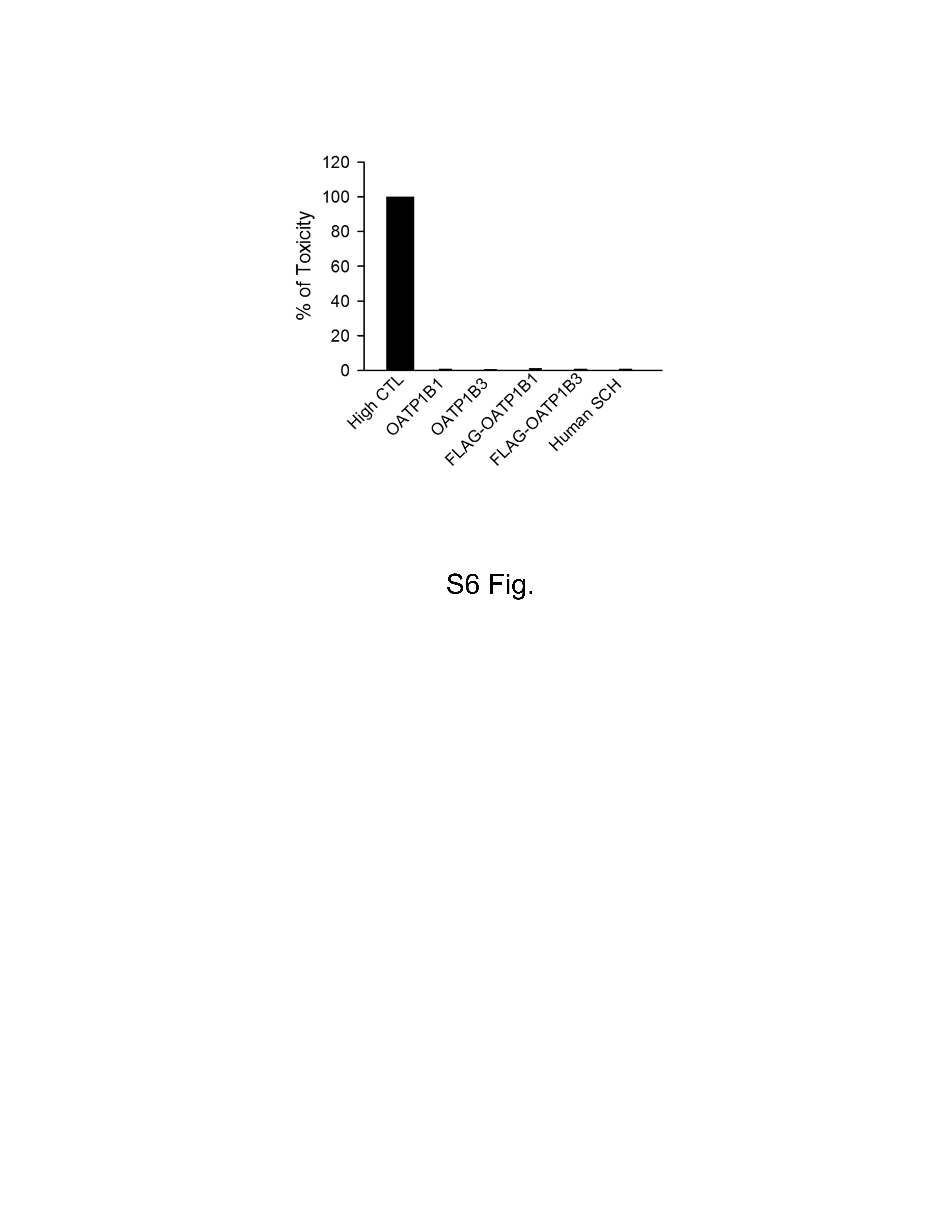

Supplement: S6 Fig — An LDH assay was performed to measure the cytotoxicity in HEK293 stable cell lines expressing OATP1B1, OATP1B3, FLAG-OATP1B1 or FLAG-OATP1B3, and human SCH treated for 7 h with 250 nM bortezomib. Triton-X (2%)-treated cells and media-treated cells served as the 100% cytotoxicity positive control and negative control, respectively. Data represents mean ± SD (n = 1 in triplicate). (TIF) [file pone.0186924.s006.tif]

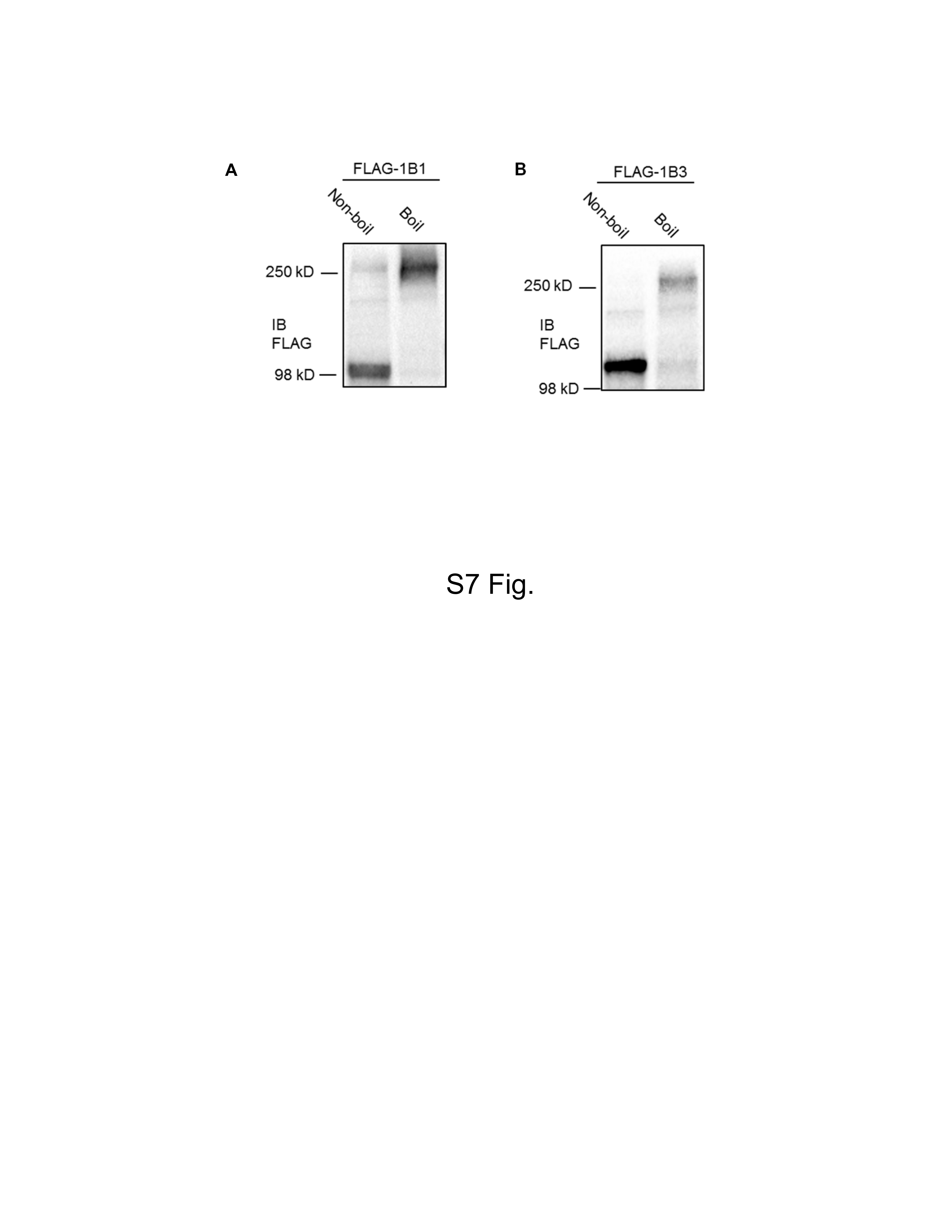

Supplement: S7 Fig — The HEK293 cells were transiently transfected with pCMV6-FLAG-OATP1B1 (A) and pCMV6-FLAG-OATP1B3 (B). Forty-eight hours after transfection, whole cell lysates were immunoblotted with FLAG antibody, with and without boiling at 100°C for 5 min. Representative images from n = 2 are shown. (TIF) [file pone.0186924.s007.tif]
